# Silymarin in non-cirrhotics with non-alcoholic steatohepatitis: A randomized, double-blind, placebo controlled trial

**DOI:** 10.1371/journal.pone.0221683

**Published:** 2019-09-19

**Authors:** Victor J. Navarro, Steven H. Belle, Massimo D’Amato, Nezam Adfhal, Elizabeth M. Brunt, Michael W. Fried, K. Rajender Reddy, Abdus S. Wahed, Stephen Harrison, on behalf of the Silymarin in NASH and C Hepatitis (SyNCH) Study Group

**Affiliations:** 1 Department of Digestive Disease and Transplantation, Einstein Medical Center and Sidney Kimmel Medical College, Philadlephia, Pennsylvania, United States of America; 2 Department of Epidemiology, University of Pittsburgh Graduate School of Public Health, Pittsburgh, Pennsylvania, United States of America; 3 Clinical Research, Rottapharm Biotech, Monza MB, Italy; 4 Division of Hepatology, Department of Medicine, Beth-Israel Deaconess Medical Center, Boston, Massachusetts, United States of America; 5 Department of Pathology and Immunology, Washington University School of Medicine, CB 8118, St. Louis, Missouri, United States of America; 6 Department of Medicine, Division of Gastroenterology and Hepatology, Liver Center, University of North Carolina, Chapel Hill, North Carolina, United States of America; 7 Department of Medicine, Division of Gastroenterology University of Pennsylvania, Philadelphia, Pennsylvania, United States of America; 8 Department of Medicine, Division of Gastroenterology, Brooke Army Medical Center, Fort Sam Houston, Texas, United States of America; Hvidovre Hospital, DENMARK

## Abstract

The botanical product silymarin, an extract of milk thistle, is commonly used by patients to treat chronic liver disease and may be a treatment for NASH due to its antioxidant properties. We aimed to assess the safety and efficacy of higher than customary doses of silymarin in non-cirrhotic patients with NASH. This exploratory randomized double-blind placebo controlled multicenter Phase II trial tested a proprietary standardized silymarin preparation (Legalon^®^, Rottapharm|Madaus, Mylan) and was conducted at 5 medical centers in the United States. Eligible adult patients had liver biopsy within 12 months showing NASH without cirrhosis with NAFLD Activity Score (NAS) ≥4 per site pathologist’s assessment. Participants were randomized to Legalon^®^ 420 mg, 700 mg, or placebo t.i.d. for 48 weeks. The primary endpoint was histological improvement ≥2 points in NAS. Of 116 patients screened, 78 were randomized. There were no significant differences in adverse events among the treatment groups. After 48–50 weeks, 4/27 (15%) in the 700 mg dose, 5/26 (19%) participants randomized to 420 mg, and 3/25 (12%) of placebo recipients reached the primary endpoint (p = 0.79) among all randomized participants, indicating no benefit from silymarin in the intention to treat analysis Review by a central pathologist demonstrated that a substantial number of participants (49, 63%) did not meet histological entry criteria and that fibrosis stage improved most in the placebo treated group, although not significantly different from other groups. Silymarin (Legalon^®^) at the higher than customary doses tested in this study is safe and well tolerated. The effect of silymarin in patients with NASH remains inconclusive due to the substantial number of patients who entered the study but did not meet entry histological criteria, the lack of a statistically significant improvement in NAS of silymarin treated patients, and the unanticipated effect of placebo on fibrosis indicate the need for additional clinical trials.

Trial Registration: clinicaltrials.gov, Identifier: NCT00680407.

## Introduction

Non-alcoholic fatty liver disease (NAFLD) and its progressive form, non-alcoholic steatohepatitis (NASH) characterized by steatosis and necroinflammation, with or without centrilobular fibrosis, have emerged as prevalent problems in Western populations. The main risk factors for developing NAFLD and NASH are components of the metabolic syndrome; increased weight, insulin resistance, hypertension, and hyperlipidemia. Liver biopsy remains the gold standard for diagnosing and assessing the degree of injury in NASH [1,2]. Although exercise and weight loss, surgical bariatric procedures, and pharmacological interventions, including insulin sensitizing agents and Vitamin E, have shown promise in treating people with NAFLD or NASH, there is no approved therapy for these disorders. There is ongoing public health concern since it is estimated that up to 30% of the US population has NAFLD, and another one third has NASH.

Silymarin, an extract of milk thistle (*Silybum marianum*), is the botanical treatment most commonly used for liver disorders in the United States, owing to its purported hepatoprotective properties [3]. Through its antioxidant properties, silymarin may mitigate lipid peroxidation and the production of free radical injury, a suspected mechanism of liver injury in NASH. Studies evaluating the use of silymarin in this capacity have found it to be effective in scavenging hydroxyl radicals, preventing the release of TNF alfa, and restoring normal levels of superoxide dismutase, a precursor of glutathione [4,5,6,7].

Recently, Kheong and colleagues reported that Silymarin at a single dose was safe and well tolerated in a Malayasian NASH population, but did not result in a statistically significant reduction in the NAS compared with placebo.[8] In view of the limited data available on dosing and pharmacokinetics of silymarin, an initial dose-ranging study [9] was performed to identify adequate silymarin doses to be tested in proof of concept studies, including the current trial. Here, we aimed to confirm the safety and preliminarly assess the efficacy of silymarin in patients with biopsy confirmed NASH without cirrhosis.

## Materials and methods

### Trial design

The “Silymarin in NASH and C Hepatitis (SyNCH)” study was a randomized, double-blind, placebo controlled phase II multicenter trial to evaluate the safety and explore the efficacy of 2 doses of a standardized form of silymarin (Legalon^®^, Rottapharm|Madaus, Mylan) compared with placebo. Although the SyNCH study comprised two patient poulations, those with Hepatitis C [10] and NASH, the current study pertains only to the latter population. Diabetic and non-diabetic patients with NASH and without cirrhosis were randomized to 3 treatment groups for up to 48-50-weeks treatment duration. Enrollment began in May 2008, and was completed in August 2011, with follow-up completed in November 2012. The trial was initially funded as a cooperative agreement award between the National Center for Complementary and Alternative Medicine and the National Institutes for Diabetes, Digestive and Kidney Diseases. However, in May 2009, funding of the study was transferred to the sponsor, Rottapharm|Madaus, as an investigator-initiated clinical trial.

### Participants

Patients over 18 years of age with AST or ALT > 40 IU/L within one year of screening and at least once during a 30-day screening period, and with suspected NAFLD were eligible for the study. At screening, patients were counseled to follow a healthy diet and lifestyle. Dietary recommendations included a decrease in saturated fats as well as total fats to <30% of total calories and macronutrient distribution of 45 to 55% carbohydrate, 25 to 35% fat and 15 to 20% protein. Patients were provided with dietary counseling to maintain glycemic control as well as to maintain a target weight/BMI that reflects no more than a +/- 10% change of body weight. Liver biopsy within 12 months of randomization confirming NASH was required for entry; the absence of cirrhosis and a NAFLD Activity Score (NAS) of 4 or greater on the baseline biopsy as read by a site pathologist qualified patients for the study. Patients meeting study entry criteria were stratified by the presence or absence of diabetes.

Patients were excluded if they had evidence for other chronic liver diseases or decompensation, a history of immunologically mediated liver disease or other severe medical illnesses, if they refused to adhere to limitations on alcohol consumption (average alcohol consumption of more than 1 drink per day or more than 2 drinks on any one day over the 30 days prior to the screening period), were diabetic with any change in anti-diabetic medication during the screening period, or had poor control of their diabetes as indicated by HbA1c > 8%. Secretagogues (sulfonylureas) and metformin were not permitted, given their purported impact on NAFLD, and anti-hyperlipidemics were permitted. In addition, patients with BMI > 45 kg/m^2^ were excluded, and weight must have been shown to be stable, with no more than a 10% change between the baseline liver biopsy and enrollment. Patients were ineligible if they had used other milk thistle preparations for a period of 90 consecutive days or longer between biopsy and initial screening, or within 30 days prior to screening if the liver biopsy was performed during the screening period. Patients were also excluded if they had used other antioxidants such as vitamin E, vitamin C, glutathione, alpha-tocopherol, or non-prescribed complementary alternative medications (including dietary supplements, megadose vitamins, herbal preparations, and special teas) within 30 days prior to screening. Medications known to produce a NAFLD or NASH-like histological picture (eg; methotrexate) were not permitted.

Participants were recruited at 5 clinical sites in the United States. The study was approved by the institutional review boards at Thomas Jefferson University, Beth Israel Deaconness Medical Center, University of North Carolina-Chapel Hill, University of Pennsylvania, the Brooke Army Medical Center, and at the Data Coordinating Center (DCC, University of Pittsburgh). All patients provided written informed consent. An independent Data and Safey Monitoring Board (DSMB) approved the initial protocol and regularly reviewed the study progress.

### Interventions and outcomes assessment

Participants were randomly assigned by the Data Coordinating Center at the University of PIttsburgh to 1 of 3 treatment groups: Legalon^®^ 420 mg or 700 mg, or placebo administered three times daily for up to 48–50 weeks. Legalon^®^ is a proprietary milk thistle seed extract standardized to a silymarin content of 140 mg per capsule. Treatment could be extended for up to 54 weeks following randomization to ensure that the post-treatment liver biopsy was performed while the participant was still taking study drug. After completing therapy, participants were monitored for an additional 12 weeks.

The doses for this study were selected based on the results of a phase I trial [9], which established safety and pharmacokinetics across a range of doses in hepatitis C and NAFLD patients. The doses, ranging from 140 to 700 mg given three times daily for 7 days, were safe and well tolerated [9]; the highest dose in the current study (700 mg three times daily) was selected as a balance between the need to achieve the highest systemic exposure and what was thought by the investigators to be a reasonable pill burden.

The primary outcome measure for efficacy was a reduction in the NAFLD Activity Score (NAS) by at least 2 points after the 48-week treatment period. The primary efficacy Intention-To-Treat (ITT) analysis was conducted based on interpretation of the baseline and end-of-treatment biopsies by a central pathologist (EB) who was masked with respect to timing and treatment group.

Secondary outcomes included reduction in the NAS by 1 point; improvement in the fibrosis stage; changes from baseline, normalization, and reduction by 50% in the serum ALT and AST; and decrease from baseline in HOMAr (determined through the formula: (Glucose mg/dL x 0.05551) x Insulin mcUI/mL 22.5) values. The primary outcome variable for safety was the occurrence of a dose-limiting toxicity during the treatment period. The toxicity rate for each dose group was calculated as the percent of subjects with an adverse event considered to be related to the study drug and resulted in a reduction or interruption of drug dose.

Following screening and randomization visits, participants were seen at weeks 2, 4, 12, 16, 24, 32, 40 and 48–54 throughout the treatment period, and followed for 12 additional weeks after therapy discontinuation. Liver biopsies were performed at the end of treatment, while the participant was still taking study medication.

Biopsies were scored by site pathologists according to the Nonalcoholic Steatohepatitis Clinical Research Network proposal for clinical trials for activity (the Nonalcoholic fatty liver disease Activity Score, NAS) and fibrosis stage.[2] NAS consists of scores for steatosis (0–3), ballooning (0–2) and lobular inflammation (0–3). Fibrosis stages are descriptive of location: zone 3 perisinusoidal (stages 1a, 1b) or periportal (1c); both zone 3 perisinusoidal and periportal (stage 2); bridging between any vascular structure (stage 3); cirrhosis (stage 4). Diagnosis of steatohepatitis (NASH diagnosis) was based on criteria published by Brunt et al. [11] The diagnosis consists of the presence of macrovesicular steatosis, hepatocyte ballooning and lobular inflammation. Fibrosis, scored separately, is not necessary for the diagnosis.

Adverse events were ascertained at each study visit, as were complete blood counts, serum biochemistries and liver tests, and urine pregnancy tests in female participants. Adherence to study medication was assessed using a summary of missed dose information obtained from patient diaries and dose counts.

### Randomization scheme

Adaptive allocation was used to minimize the imbalance among treatment arms. Participants were allocated to treatment arm within strata defined by site and diabetes status, by means of a web-based system. Participants, investigators, clinical site staff and pathologists were masked to treatment assignment.

### Statistical analyses

#### Sample size determination

Assuming a NAS reduction of at least 2 points in 15% of participants in the placebo arm (most likely to occur due to biopsy sampling error or misclassification or because of the lifestyle change) versus an average of 47.5% of participants taking silymarin (e.g. 40% and 55% in the lower and higher dose group, respectively), 26 participants in each treatment group would provide 80% power to reject the null hypothesis at α = 0.10 using a Chi-square test with 2 degrees of freedom. Per DSMB suggestion, the last observation carried forward method was used to impute the final outcome if a follow-up liver biopsy prior to the end of treatment biopsy was available, otherwise participants without a post baseline biopsy were considered treatment failures. Thus, no over-recruitment to account for loss to follow-up was necessary.

#### Statistical methods

Statistical analyses for the primary efficacy assessments were carried out on the Intention-To-Treat (ITT) population, defined as all the randomized patients. Notably, upon review of all liver biopsies by the central pathologist during the efficacy analysis, a proportion of pre-randomization liver biopsies did not meet entry histological criteria. Therefore, a supplementary analysis was performed with the subgroup of participants whose biopsies met entry histological criteria per the central pathologist.

Baseline characteristics across treatment groups are presented using frequencies and percents for categorical variables, using means and standard deviations for symmetric continuous variables, and using medians and percentiles for skewed continuous variables. By randomization scheme, they were supposed to be similar across groups and hence formal comparisons across treatment groups for these variables were performed only for verification purpose with a chi-square test or its exact version for categorical measures, and with parametric (F-test) or non-parametric (Kruskal-Wallis test) ANOVA for continuous measures.

The primary and secondary efficacy binary outcomes are reported using frequencies and percentages and compared across groups using the Chi-square test or its exact version, as appropriate. Missing NAS scores for participants who dropped out of the study were imputed using the last observation carried forward (LOCF) if a post-baseline liver biopsy was available at an earlier visit. Otherwise the drop out was considered to be a treatment failure (did not meet the endpoint). Subjects with baseline or end of treatment biopsy that could not be evaluated by the central pathologist were also considered as treatment failures.

For continuous secondary efficacy outcomes, changes from baseline were reported using means and standard deviations or medians and percentiles, as appropriate, and compared among treatment groups by means of a one-way ANalysis Of Variance (ANOVA) using an F-test or Kruskal-Wallis test, as appropriate. Toxicity rate and the incidence of AEs during the study were compared among treatment groups using a Chi-square test or its exact counterpart.

SAS version 9.3 (SAS Institute Inc., Cary, NC) was used for statistical analyses.

All authors had access to the study data and had reviewed and approved the final manuscript.

## Results

**Fig 1:**
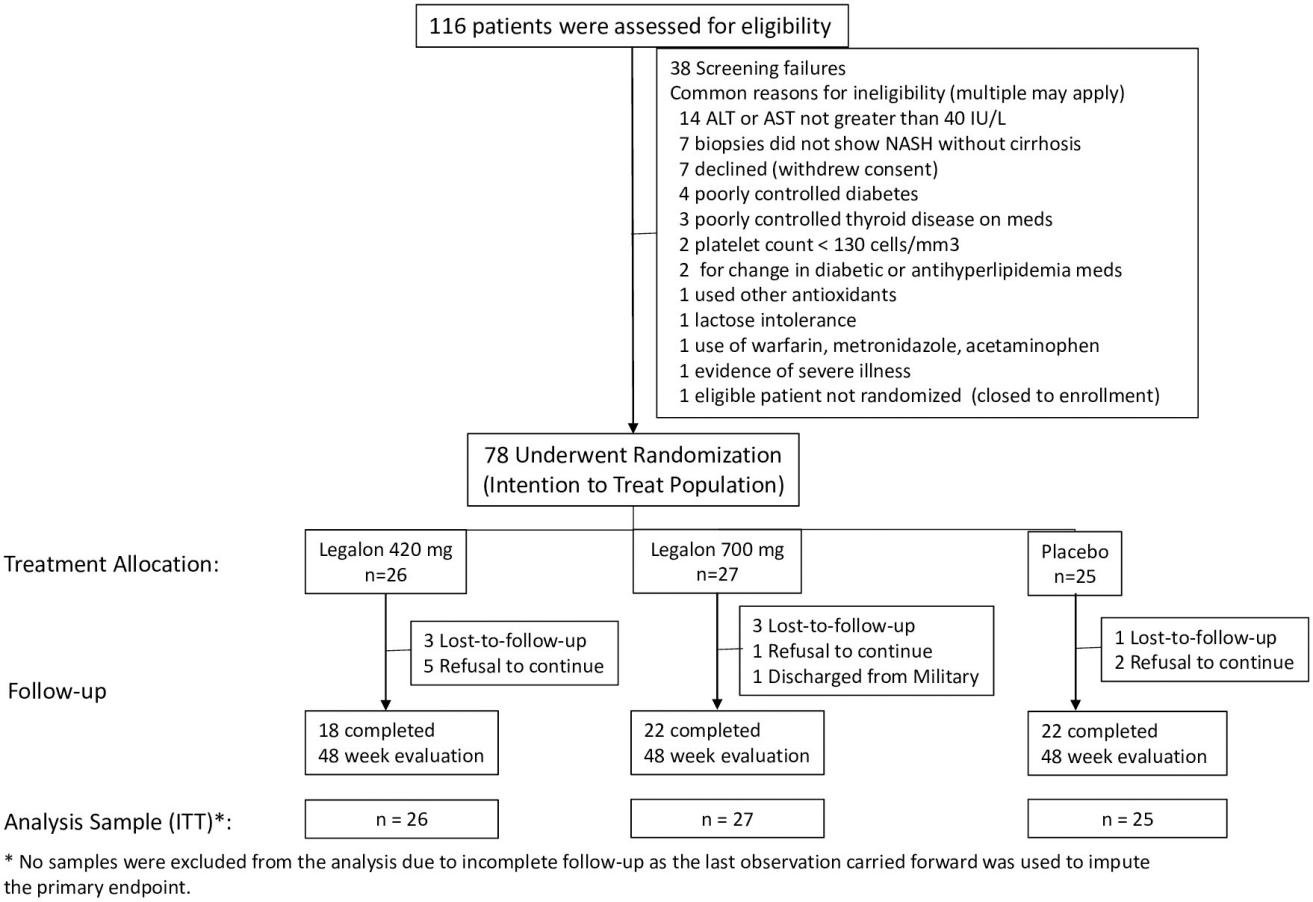
Displayed is patient enrollment. Of the 116 patients assessed for eligibility, 38 failed screening, and 78 underwent randomization (25 to Placebo, 26 to 420 mg, and 27 to 700 mg); this group comprised the intention to treat study population. Twenty-nine of the 78 randomized patients actually met histological criteria for NASH, as determined by the study pathologist (BB). Specifically, 34 biopsies showed an NAS < 4 or no NASH; 1 showed NASH with cirrhosis; and 14 biopsies were either unavailable or not evaluable. Therefore, a subgroup analysis was conducted on the 29 patients, referred to as the intended target population.

A total of 116 patients were screened for enrollment: 78 were randomized; 26 to the Legalon^®^ 420 mg treatment arm, 27 to the Legalon^®^ 700 mg treatment arm and 25 to the placebo treatment arm. Among the 38 patients who were not randomized, the most common reasons were an ALT or AST not greater than 40 IU/L (14 patients), liver biopsy not demonstrating features consistent with NASH without cirrhosis as determined by the site pathologist (7 patients), and withdrawal of consent (7 patients). Fig 1 illustrates patient enrollment.

The baseline demographic, clinical, laboratory and histological (NAS) characteristics of the three treatment groups were similar in the ITT population, as shown in Table 1. Participants had median age of 48.3 years, median BMI of 34.1 kg/m^2^, were predominantly male (58%), of white race (95%), and non-Hispanic (83%).

**Table 1:** Baseline characteristics of the study population.

	Legalon^®^ 420 mg (N = 26)	Legalon^®^ 700 mg (N = 27)	Placebo (N = 25)	Total (N = 78)
**Demographics**				
Age, years (median)	47.3 (10.8)	48.2 (11.4)	49.5 (10.9)	48.3 (10.9)
Gender				
Female	13 (50%)	9 (33%)	11 (44%)	33 (42%)
Male	13 (50%)	18 (67%)	14 (56%)	45 (58%)
Race (White)	25 (96%)	27 (100%)	22 (88%)	74 (95%)
Ethnicity (Hispanic)	5 (19%)	5 (18%)	3 (12%)	13 (17%)
Diabetes (stratum)				
Yes	6 (23%)	8 (30%)	7 (28%)	21 (27%)
No	20 (77%)	19 (70%)	18 (72%)	57 (73%)
**Laboratory examinations and metabolic factors**				
Platelets (x10^3^ cells/mm^3^)	246 (66)	250 (60)	229 (51)	242 (59)
ALT (IU/L)	80(66,111)	61(51,94)	65(45,108)	70(53,101)
AST (IU/L)	57(43,71)	46(36,58)	51(37,62)	52(39,63)
Alkaline Phosphatase (IU/L)	64(56,84)	67(59,77)	82(61,96)	69(59,88)
Triglycerides (mg/dL)	130(119,173)	153(101,197)	153(114,179)	150(110,189)
Cholesterol (mg/dL)	191 (42)	175 (36)	174 (38)	180 (39)
Fasting glucose (mg/dL)	99(86,106)	98(87,105)	101(93, 127)	98(87,115)
HOMAr	4.9(3.4,7.4)	4.5(2.9,9.0)	5.5(3.1,9.1)	5.0(3.0,8.4)
BMI (kg/m^2^)	35.3 (4.8)	33.5 (4.3)	33.4 (4.8)	34.1 (4.7)
**NAS**				
Site pathologist	4.9 (1.1)	4.8 (0.9)	4.6 (1.0)	4.8 (1.0)
Central pathologist §	4.4 (1.7)	4.4 (1.7)	4.4 (1.3)	4.4 (1.6)
**Alcohol use**				
≥ 7 alcoholic beverages/day at least once in the past 12 months	2 (8)	2 (7)	3 (12)	7 (9)
**Patient reported outcomes**				
SF-36				
Physical component	46.8 (9.4)	43.4 (9.5)	48.8 (8.3)	46.3 (9.2)
Mental component	53.6 (4.9)	51.7 (8.5)	54.1 (5.6)	53.1 (6.6)
CES-D	16.4 (5.3)	16.8 (3.6)	15.3 (5.8)	16.2 (4.9)
CLDQ	5.4 (0.9)	5.2 (0.8)	5.4 (1.0)	5.3 (0.9)

Data are n (%) for categorical variables, mean (SD) for symmetric and median(25^th^ percentile, 75^th^ percentile) for skewed continuous variables.

^§^ NAS by central pathologist was missing in 3, 5 and 4 patients in the Legalon^®^ 420 mg, Legalon^®^ 700 mg and placebo groups, respectively.

CES-D: Center for Epidemiologic Studies Depression questionnaire. CLDQ: Chronic Liver Disease Questionnaire.

After 48–50 weeks of treatment, 4 participants of 27 (15%) in the 700 mg dose group, 5 of 26 (19%) in the 420 mg dose group, and 3 of 25 (12%) in the placebo group reached the primary endpoint of at least 2-point reduction in the NAS (p = 0.79; Table 2). Thus, there were no statistically significant differences among the treatment groups in the ITT analysis. Sixty two (80%) participants completed the study; 18/26 (69%) in the Legalon^®^ 420 mg dose group, 22/27 (82%) in the Legalon^®^ 700 mg dose group and 22/25 (88%) in the placebo group. Of the 16 participants who discontinued the study, the most common reasons for withdrawal were refusal to continue and loss to follow-up.

**Table 2:** Analysis of primary and secondary efficacy outcome measures.

	Legalon^®^ 420 mg	Legalon^®^ 700 mg	Placebo	P values
ITT population	(N = 26)	(N = 27)	(N = 25)	
**Primary endpoint**				
≥2 NAS point reduction	5 (19%)	4 (15%)	3 (12%)	0.79
**Secondary endpoints**				
≥1 NAS improvement	8 (31%)	7 (26%)	6 (24%)	0.85
ALT normalized °	2 (8%)	6 (25%)	1 (5%)	0.08
AST normalized °	4 (18%)	7 (37%)	6 (35%)	0.39
HOMAr decreased	14 (54%)	13 (48%)	11 (44%)	0.88
Fibrosis stage improved	3 (12%)	7 (26%)	7 (28%)	0.30

Data are n (%).

° Percentages calculated on patients with abnormal value (>40 IU/L) at baseline: Upper panel: ALT N = 25, 24 and 21 in the Legalon^®^ 420 mg, Legalon^®^ 700 mg and placebo groups, respectively; Upper panel: AST N = 22, 19 and 17 in the Legalon^®^ 420 mg, Legalon^®^ 700 mg and placebo groups, respectively; Lower panel: ALT N = 10, 8 and 10 in the Legalon^®^ 420 mg, Legalon^®^ 700 mg and placebo groups, respectively; Lower panel: AST N = 9, 7 and 8 in the Legalon^®^ 420 mg, Legalon^®^ 700 mg and placebo groups, respectively.

The analysis for secondary endpoints in the ITT population showed no statistically significant differences with respect to NAS improvement (at least 1 point), normalization of the ALT or AST, change in the HOMAr, and improvement in fibrosis stage (Table 2). A relatively higher percentage of participants in the Legalon^®^ 420 mg (23%) and Legalon^®^ 700 mg (19%) dose groups showed an improvement in steatosis than in the placebo group (16%), but the difference was not statistically significant. No statistically significant differences among treatment groups were observed for the other NAS components (Table 3).

**Table 3:** Hepatic histologic scores. Intention to Treat population.

Histologic Feature	Legalon^®^ 420 mg (N = 26)*		Legalon^®^ 700 mg (N = 27)°		Placebo (N = 25)^#^	
	Before Treatment	After Treatment	Before Treatment	After Treatment	Before Treatment	After Treatment
**NAS**						
Patients with a reduction in score of ≥2 –no./total no. (%)		5/26 (19%)		4/27 (15%)		3/25 (12%)
Patients with any improvement in score–no./total no. (%)		8/26 (31%)		7/27 (26%)		6/25 (24%)
**Steatosis**						
Score–no. of patients						
0 (<5%)	0	0	1	1	1	0
1 (5–33%)	9	7	7	9	7	8
2 (>33–66%)	9	7	8	5	9	9
3 (>66%)	6	3	7	7	4	3
Patients with any improvement in score–no./total no. (%)		6/26 (23%)		5/27 (19%)		3/25 (12%)
**Hepatocyte ballooning**						
Score–no. of patients						
0 (None)	10	6	10	11	8	7
1 (Few)	5	5	6	3	6	7
2 (Many)	8	6	7	8	7	6
Patients with any improvement in score–no./total no. (%)		4/26 (15%)		5/27 (19%)		4/25 (16%)
**Lobular inflammation**						
Score–no. of patients						
0 (no foci)	1	2	1	1	0	1
1 (<2 foci per 200x field)	11	7	10	12	8	11
2 (2–4 foci per 200x field)	8	7	7	8	11	7
3 (>4 foci per 200x field)	3	1	4	1	2	1
Patients with any improvement in score–no./total no. (%)		5/26 (19%)		6/27 (22%)		5/25 (20%)
**Fibrosis**						
Score–no. of patients						
0 (None)	2	2	4	6	5	6
1 (Perisinusoidal or periportal)	12	5	8	5	9	8
2 (Perisinusoidal and portal/periportal)	3	2	5	5	2	3
3 (Bridging fibrosis)	5	5	5	3	4	3
4 (Cirrhosis)	1	2	1	3	0	0
Patients with any improvement in score–no./total no. (%)		3/26 (12%)		7/27 (26%)		7/25 (28%)

* The pre-treatment biopsy was considered not evaluable for 2 patients in regard to steatosis, and for 3 patients in regard to hepatocyte ballooning, lobular inflammation and fibrosis. The post-treatment biopsy was considered not evaluable for 1 patient in regard to steatosis, hepatocyte ballooning and lobular inflammation, and for 2 patients in regard to fibrosis, whereas for 8 patients the post-treatment biopsy was not available.

° The pre-treatment biopsy was considered to be not evaluable for 4 patients in regard to steatosis, hepatocyte ballooning and fibrosis and for 5 patients in regard to lobular inflammation. The post-treatment biopsy was not available for 5 patients.

^#^ The pre-treatment biopsy was considered to be not evaluable for 1 patient in regard to steatosis, hepatocyte ballooning and lobular inflammation, and for 2 patients in regard to fibrosis, whereas for 3 patients the pre-treatment biopsy was not available to the central pathologist. The post-treatment biopsy was not available for 4 patients, whereas for 1 patient it was considered to be not evaluable.

As stated previously, upon review of the biopsies by the central pathologist, a large proportion of entry biopsies (63%) did not meet histological entry criteria, despite having been scored differently by site pathologists. Specifically, 34 of the 78 participants (43.6%) had biopsies showing NAS <4 or histological diagnosis criteria for NASH that were not met; 1 participant (1.3%) had cirrhosis; and 14 participants (17.9%) had pre-treatment biopsies that were either not available to the central pathologist or considered to be not evaluable due to insufficient tissue. Thus, the remaining sample comprised 10 in the 420 mg dose group, 9 in the 700 mg dose group, and 10 in the placebo arm. There were 4 participants of 9 (44.4%) in the 700 mg dose group, 3 of 10 (30%) in the 420 mg dose group, and 1 of 10 (10%) in the placebo group who reached the primary endpoint of at least 2-point reduction in the NAS (p = 0.27; S1 Table). Analysis of the hepatic histologic score changes showed that more participants assigned to Legalon^®^ 420 mg and Legalon^®^ 700 mg compared to placebo had an improvement in steatosis and lobular inflammation, but the improvements were not statistically significant (S2 Table). As far as the analyses of the other secondary endpoints in this subgroup are concerned, no meaningful changes were observed among the three treatment groups in ALT, AST, or HOMAr (S1 Table).

During the study, a total of 89 Adverse Events (AEs) were reported by 44 (56%) participants (Table 4). Gastrointestinal complaints were the most common. The incidence of AEs did not differ significantly in the three groups (p = 0.49). Four Serious Adverse Events occurred in 5% of patients, all considered not related to the study drug administration. Overall, only 2 patients experienced an adverse event that was rated as related to the study drug and resulted in a reduction or interruption of drug dose (both in the Legalon^®^ 420 mg dose group), leading to the following estimates of double-blind toxicity rates: 8% in the Legalon^®^ 420 mg dose group and 0% in Legalon^®^ 700 mg and placebo groups (p = 0.21).

**Table 4:** Adverse events by treatment arms.

AE Type	#Patients with AE [n(%)]			#AE		
	Legalon^®^ 420 mg (N = 26)	Legalon^®^ 700 mg (N = 27)	Placebo (N = 25)	Legalon^®^ 420 mg (N = 26)	Legalon^®^ 700 mg (N = 27)	Placebo (N = 25)
AEs	17 (65.4%)	15 (55.6%)	12 (48.0%)	28	28	33
Most common classes of AEs, by body system						
Gastrointestinal	5 (19.2%)	4 (14.8%)	4 (16.0%)	6	8	4
Respiratory	2 (7.7%)	3 (11.1%)	2 (8.0%)	3	3	7
Musculoskeletal	2 (7.7%)	1 (3.7%)	5 (20.0%)	2	1	5
Headache	2 (7.7%)	2 (7.4%)	2 (8.0%)	2	2	2
Cardiac	3 (11.5%)	1 (3.7%)	2 (8.0%)	3	1	2
Other	11 (42.3%)	10 (37.0%)	8 (32.0%)	12	13	13

## Discussion

Building upon the purported health benefits of milk thistle extract and ongoing interest in it as a therapeutic agent [12], this trial was designed to test whether a particular formulation of milk thistle could mitigate NASH related liver injury. In addition, the trial aimed to establish the safety of this milk thistle formulation over the range of doses used in this study, which were higher than customary. At all doses, we found that Legalon^®^ was safe and well tolerated, with no difference in adverse events among the treatment groups. This trial showed that more participants assigned to Legalon^®^ groups had an improvement in steatosis and lobular inflammation compared with placebo, but failed to show a statistically significant histological improvement of NASH.

No meaningful changes were observed among the three treatment groups in ALT, AST or HOMAr. No significant changes were observed in other efficacy assessments, which included change from baseline in liver fibrosis; proportion of participants with transaminases returning to normal range or with a reduction greater than 50%; and improvement of insulin resistance.

Kheong et al [8], in their randomized placebo controlled trial of Silymarin 700 mg given three times a day for 48 weeks to NASH patients, likewise did not demonstrate a statistically significant reduction in the NAS; ≥ 30% was targeted as the primary outcome. However, the treatment arm was associated with reductions in hepatic fibrosis by histology, and liver stiffness by transient elastography. The study was similar in design and duration of treatment as the current study, although only one dose was used. The majority of patients (89%) underwent post-treatment biopsy.

The current study excluded cirrhotic patients for several reasons, and thus the findings cannot be generalized to this population. Most importantly, histology of cirrhotics with suspected NASH often does not reflect the typical features of steatosis and steatohepatitis. Therefore, the diagnosis of NASH induced cirrhosis would have been based on circumstantial evidence, such as the history of insulin resistance or obesity. Such assumptions of disease causation without histological confirmation would have allowed for potential misclassification bias. Moreover, the primary endpoint of histological improvement could not have been assessed in this population. Furthermore, patients with cirrhosis demonstrate pharmacokinetics which differ from those without cirrhosis.

A limitation of the current study that is relevant to the primary endpoint and that may also limit generalizability is the large number of randomized patients ultimately found by the central pathologist to have liver biopsies that did not meet histologic inclusion criteria. This motivated an additional analysis of those participants for whom the liver biopsies met inclusion criteria, as judged by the central pathologist (EB). However, though this supplementary analysis showed a trend in the point estimates of patients reaching the primary endpoint consistent with the efficacy hypothesis defined in the protocol, the sample of the intended target population was too small to draw an adequately powered conclusion regarding efficacy. Importantly, the reduction in the NAS score observed in the intended target population of this study in the active and control arms are in line with those observed in the active and placebo arms and of a recently published trial.[13] This point notwithstanding, the fact remains that silymarin treatment in this study showed no improvement in the NAS in the ITT population. Furthermore, the large proportion of patients who failed to meet the histological entry criteria combined with a statistically non-significant improvement in fibrosis in the placebo group indicate the need for additional clinical trials.

In conclusion, Legalon^®^ at the higher than customary doses tested in this study is safe and well tolerated. Additional studies are warranted, ideally using improved methods to diagnose and grade NASH. At a minimum, future trials that rely on hiostological endpoints ought to make accommodations for optimizing liver biopsy samples.

## Supporting information

S1 Table Analysis of primary and secondary efficacy outcome measures in the patients who met histological inclusion criteria. (DOC)

S2 Table Hepatic histologic scores in patients who met histological inclusion criteria. (DOC)

S1 File Study protocol. (PDF)

S2 File Consort 2010 checklist. (DOC)

## Abbreviations

ALT: Alanine Aminotransferase
ANOVA: Analysis of Variance
AST: Aspartate Aminotransferase
DSMB: Data and Safety Monitoring Board
HOMAr: Homeostatic Model Assessment
LOCF: Last Observation Carried Forward
NAFLD: Non-Alcoholic Fatty Liver Disease
NAS: Non-Alcoholic Fatty Liver Disease Activity Score
NASH: Non-Alcoholic Steatohepatitis
