# Correction: Silymarin in non-cirrhotics with non-alcoholic steatohepatitis: A randomized, double-blind, placebo controlled trial

**DOI:** 10.1371/journal.pone.0223915

**Published:** 2019-10-10

**Authors:** Victor J. Navarro, Steven H. Belle, Massimo D’Amato, Nezam Afdhal, Elizabeth M. Brunt, Michael W. Fried, K. Rajender Reddy, Abdus S. Wahed, Stephen Harrison, on behalf of the Silymarin in NASH and C Hepatitis (SyNCH) Study Group

The fourth author’s name is incorrect. The correct name is Nezam Afdhal. The correct citation is: Navarro VJ, Belle SH, D’Amato M, Afdhal N, Brunt EM, Fried MW, et al. (2019) Silymarin in non-cirrhotics with non-alcoholic steatohepatitis: A randomized, double-blind, placebo controlled trial. PLoS ONE 14(9): e0221683. https://doi.org/10.1371/journal.pone.0221683

## References

[1] Navarro VJ, Belle SH, D’Amato M, Adfhal N, Brunt EM, Fried MW, et al (2019) Silymarin in non-cirrhotics with non-alcoholic steatohepatitis: A randomized, double-blind, placebo controlled trial. PLoS ONE 14(9): e0221683 10.1371/journal.pone.0221683 31536511 PMC6752871

