# Supplementary material for: Silymarin in non-cirrhotics with non-alcoholic steatohepatitis: A randomized, double-blind, placebo controlled trial
Source: PLoS One. 2019 Sep 19;14(9):e0221683. doi: 10.1371/journal.pone.0221683 (PMC6752871; doi:10.1371/journal.pone.0221683)
Supplement: S1 Table — (DOC) [file pone.0221683.s001.doc]

**SUPPLEMENTARY MATERIALS**

**Table 1. Analysis of primary and secondary efficacy outcome measures in the patients who met histological inclusion criteria.**

| **Patients who met histological inclusion criteria** | **(N=10)** | **(N=9)** | **(N=10)** |  |
| --- | --- | --- | --- | --- |
| **Primary endpoint** |  |  |  |  |
| ≥2 NAS point reduction | 3 (30%) | 4 (44%) | 1 (10%) | 0.27 |
| **Secondary endpoints** |  |  |  |  |
| ≥1 NAS improvement | 5 (50%) | 5 (56%) | 3 (30%) | 0.64 |
| ALT normalized ° | 1 (10%) | 2 (25%) | 1 (10%) | 0.65 |
| AST normalized ° | 1 (11%) | 3 (43%) | 3 (38%) | 0.33 |
| HOMAr decreased | 5 (50%) | 5 (56%) | 4 (40%) | 0.80 |
| Fibrosis stage improved | 1 (10%) | 3 (33%) | 3 (30%) | 0.56 |
| Data are n (%).  ° Percentages calculated on patients with abnormal value (>40 IU/L) at baseline:  Upper panel: ALT N=25, 24 and 21 in the Legalon® 420 mg, Legalon® 700 mg and placebo groups, respectively;  Upper panel: AST N=22, 19 and 17 in the Legalon® 420 mg, Legalon® 700 mg and placebo groups, respectively;  Lower panel: ALT N=10, 8 and 10 in the Legalon® 420 mg, Legalon® 700 mg and placebo groups, respectively;  Lower panel: AST N=9, 7 and 8 in the Legalon® 420 mg, Legalon® 700 mg and placebo groups, respectively. | | | |  |
