# Supplementary material for: Silymarin in non-cirrhotics with non-alcoholic steatohepatitis: A randomized, double-blind, placebo controlled trial
Source: PLoS One. 2019 Sep 19;14(9):e0221683. doi: 10.1371/journal.pone.0221683 (PMC6752871; doi:10.1371/journal.pone.0221683)
Supplement: S2 Table — (DOC) [file pone.0221683.s002.doc]

**SUPPLEMENTARY MATERIALS**

**Table 2. Hepatic histologic scores in patients who met histological inclusion criteria.**

| **Histologic Feature** | **Legalon® 420 mg**  **(N=10)** | | **Legalon® 700 mg**  **(N=9)** | | **Placebo**  **(N=10)** | |
| --- | --- | --- | --- | --- | --- | --- |
|  | Before Treatment | After^*,°^ Treatment | Before Treatment | After^*^ Treatment | Before Treatment | After^*^ Treatment |
| **NAS** |  |  |  |  |  |  |
| Patients with a reduction in score of ≥2 – no./total no. (%) |  | 3/10 (30%) |  | 4/9  (44%) |  | 1/10 (10%) |
| Patients with any improvement in score – no./total no. (%) |  | 5/10 (50%) |  | 5/9  (56%) |  | 3/10 (30%) |
| **Steatosis** |  |  |  |  |  |  |
| Score –no. of patients |  |  |  |  |  |  |
| 0 (<5%) | 0 | 0 | 0 | 0 | 0 | 0 |
| 1 (5-33%) | 2 | 3 | 2 | 4 | 5 | 2 |
| 2 (>33-66%) | 5 | 3 | 4 | 1 | 4 | 5 |
| 3 (>66%) | 3 | 2 | 3 | 2 | 1 | 1 |
| Patients with any improvement in score – no./total no. (%) |  | 3/10 (30%) |  | 3/9  (33%) |  | 1/10 (10%) |
| **Hepatocyte ballooning** |  |  |  |  |  |  |
| Score –no. of patients |  |  |  |  |  |  |
| 0 (None) | 0 | 2 | 0 | 3 | 0 | 1 |
| 1 (Few) | 3 | 1 | 4 | 1 | 4 | 2 |
| 2 (Many) | 7 | 5 | 5 | 3 | 6 | 5 |
| Patients with any improvement in score – no./total no. (%) |  | 3/10 (30%) |  | 4/9  (44%) |  | 3/10 (30%) |
| **Lobular inflammation** |  |  |  |  |  |  |
| Score –no. of patients |  |  |  |  |  |  |
| 0 (no foci) | 0 | 0 | 0 | 1 | 0 | 0 |
| 1 (<2 foci per 200x field) | 3 | 3 | 2 | 4 | 4 | 3 |
| 2 (2-4 foci per 200x field) | 5 | 4 | 5 | 2 | 4 | 4 |
| 3 (>4 foci per 200x field) | 2 | 1 | 2 | 0 | 2 | 1 |
| Patients with any improvement in score – no./total no. (%) |  | 3/10 (30%) |  | 4/9  (44%) |  | 2/10 (20%) |
| **Fibrosis** |  |  |  |  |  |  |
| Score –no. of patients |  |  |  |  |  |  |
| 0 (None) | 0 | 0 | 0 | 2 | 1 | 0 |
| 1 (Perisinusoidal or  periportal) | 3 | 3 | 3 | 1 | 4 | 5 |
| 2 (Perisinusoidal and  portal/periportal) | 2 | 0 | 3 | 1 | 1 | 0 |
| 3 (Bridging fibrosis) | 5 | 2 | 3 | 1 | 4 | 3 |
| 4 (Cirrhosis) | 0 | 2 | 0 | 2 | 0 | 0 |
| Patients with any improvement in score – no./total no. (%) |  | 1/10 (10%) |  | 3/9  (33%) |  | 3/10 (30%) |
| * In 2 patients for each treatment group, the biopsy after treatment was not performed.  ° For 1 patient in the Legalon® 420 mg group, the biopsy after treatment was available but the fibrosis score was missing. | | | | | | |
