# Supplementary material for: Silymarin in non-cirrhotics with non-alcoholic steatohepatitis: A randomized, double-blind, placebo controlled trial
Source: PLoS One. 2019 Sep 19;14(9):e0221683. doi: 10.1371/journal.pone.0221683 (PMC6752871; doi:10.1371/journal.pone.0221683)
Supplement: S1 File — (PDF) [file pone.0221683.s003.pdf]

SyNCH NASH Phase II:

A Multicenter, Randomized, Double Masked, Placebo Controlled Phase II Study to Assess the Safety and Efficacy of a Standardized Orally Administered Silymarin Preparation (Legalon®) for the Treatment of Non-Cirrhotic Patients with Non-Alcoholic Steatohepatitis

IND 74,887

Principal Investigators:
Michael W. Fried, M.D.
Rajender Reddy, M.D.
Nezam Afdhal, M.D.
Victor Navarro, M.D.
Steven H. Belle, Ph.D.

Study Centers:
University of North Carolina
University of Pennsylvania
Harvard University
Thomas Jefferson University
University of Pittsburgh

Funding source:
National Center for Complementary and Alternative Medicine (NCCAM), National Institute of Diabetes and Digestive and Kidney Diseases (NIDDK), National Institutes of Health

Summary

Non-alcoholic steatohepatitis (NASH) is a variant of non-alcoholic fatty liver disease (NAFLD) and the metabolic syndrome, which is associated with diabetes and obesity. NAFLD is seen in 30 million Americans and up to 10% may have NASH, the progressive form of disease, which can lead to cirrhosis and liver cancer. No proven treatments are currently available for NASH, although weight loss and exercise are recommended. This disease represents a therapeutic unmet need and is a suitable indication for novel clinical agents. Silymarin, the active ingredient of milk thistle, has been in longstanding use as an anti-oxidant and hepatoprotective agent but has not been rigorously evaluated in clinical trials for liver disease. Clinical studies of an agent such as silymarin have been hampered by the lack of a standardized preparation and the difficulties in designing studies with homogenous populations and appropriate endpoints for the evaluation of efficacy.

This is a multicenter, randomized, double masked, placebo controlled Phase II trial to evaluate the safety and explore the efficacy of silymarin (Legalon®) compared with placebo on hepatic histology in patients with NASH after 48-50 weeks of therapy. This study is being sponsored through a cooperative agreement (U01) award from the NCCAM and the NIDDK (RFA-AT-05-006: "Phase I/II Trials of Silymarin for Chronic Liver Diseases"). The broad aim of this study is to evaluate the safety and explore the efficacy of silymarin (Legalon®) in NASH patients and to form the basis for future studies which will establish its efficacy for treating patients with NASH. The specific objectives of this study are to determine the effect of silymarin (Legalon®) on the histologic NASH Activity Score (NAS), the liver enzymes, and HOMAr. The primary endpoint of the study is an improvement in the NAS by at least 2 points. Various secondary endpoints will be assessed, including the change in liver enzymes and HOMAr. Also, pharmacokinetic assessments (population PK) will be performed to confirm phase I pharmacokinetic findings.

Following a lead-in phase, silymarin (Legalon®) will be administered to patients with histologically confirmed NASH randomized to placebo or one of two active treatment groups. One active treatment group will receive 420 mg, each dose given three times daily, the other active treatment group will receive 700 mg, each dose given three times daily. Patients will be treated for 48-50 weeks. Liver biopsy will be performed prior to, and immediately after, the treatment phase. Blood will be collected for pharmacokinetic analysis for confirming estimates of PK parameters obtained during phase I, for measuring biomarkers, and for storage for future studies.

1.0 Background

Non-alcoholic fatty liver disease and Non-alcoholic steatohepatitis

Non-alcoholic fatty liver disease (NAFLD) and non-alcoholic steatohepatitis (NASH), initially described in 1980[1], are now recognized as common disease entities. Among obese patients, 60% have steatosis, 20% to 25% have steatohepatitis, and approximately 3% have cirrhosis.[2] Obesity is one of multiple risk factors identified in the development of NAFLD/NASH. Insulin resistance may be the most important factor predisposing to fat accumulation and steatohepatitis. In addition, hypertriglyceridemia, hypertension, or a family history of the above are commonly present in patients with NAFLD/NASH.[3] Due to the increased prevalence of females with advanced NASH associated liver disease, female gender has been identified as a risk factor for disease progression. [,4,5] However, this is less well studied in the less advanced stages of the disease. A racial disparity may also exist. It has been suggested that African Americans are less likely to be affected by NAFLD/NASH, compared with European and Hispanic Americans.[6,7]

The mechanism for the progression of fatty liver to steatohepatitis has not been fully elucidated. However, a "two hit" hypothesis has been proposed in the progression of fatty liver to steatohepatitis.[8] Pre-existing conditions, such as obesity, insulin resistance, and hypertriglyceridemia comprise the "first hit" leading to the accumulation of fatty acids in the liver. Increased levels of free fatty acids lead to a cascade of oxidative injury resulting in excessive production of reactive oxygen species. When the antioxidant defense mechanisms become overwhelmed, fatty acid oxidation and lipid peroxidation ensue, comprising the "second hit", leading to the transformation of steatosis to steatohepatitis. Markers of lipid peroxidation, insulin resistance indices, and body mass index measurements have been shown to correlate with disease activity in NASH studies.

Adipose tissue also plays a significant role in the pathogenesis of NASH, through its ability to secrete various bioactive proteins which affect lipid metabolism. Two such proteins are leptin and adiponectin. Both function as hormones in the regulation of energy use, and lipid and carbohydrate metabolism.[9] Studies have shown that low levels of either one of these adipocyte hormones contributes to progressive insulin resistance and eventually the development of NASH. On the other hand, TNF-α, another adipokine secreted by adipose tissue, functions antagonistically to adiponectin and contributes to further insulin resistance, which in turn leads to steatosis, and ultimately the development of NASH. It has been suggested that an imbalance in these insulin modulating hormones may play a key role in the pathogenesis of NASH, and such markers may correlate with the severity of the disease. [,,10]

The clinical presentation of fatty liver and NASH is often characterized by asymptomatic liver test elevations in combination with the presence of fat on hepatic imaging. The diagnosis is often assumed only after a careful search fails to yield another cause for these findings. However, liver biopsy remains the gold standard for diagnosing and assessing the degree of injury in NASH.[11,12] Histologically, steatohepatitis is characterized by steatosis, hepatocyte ballooning, and acute and chronic lobular inflammation. Other features include the presence of Mallory's hyaline and glycogenated nuclei.[13] A scoring system has been proposed for steatohepatitis so that disease progression can be followed in a consistent manner and endpoints in study protocols can be universalized. Most recently Kleiner et al proposed a scoring system to include only those features defined in the scoring system that are potentially reversible, that is, steatosis, lobular inflammation, and ballooning. Although validated, this system has not been assessed in the context of a clinical trial for its performance

characteristics. It should be noted that even histology has its limitations. Significant sampling variability has been shown; thus a combination of both histology and alternative markers of disease and inflammation should be used in following disease progression or improvement in NASH.[14] Additionally, cross-sectional imaging studies, such as lipid-sensitive (chemical shift) MRI, can show if steatosis or other liver parenchymal abnormalities show regional or geographic variability.

Despite an increasing understanding of the pathophysiology underlying steatohepatitis, a proven effective therapy has not been found. Generally, treatment entails mitigating risk factors, such as obesity, insulin resistance, and hyperlipidemia. Drugs that increase insulin sensitivity, specifically the thiazolidinediones, are being studied as therapeutic options for steatohepatitis. These agents serve as PPAR-γ receptor ligands, thereby facilitating insulin responsiveness and reducing the need for hepatic gluconeogensis.[,15] Two recent studies evaluated pioglitazone and rosiglitazone as therapeutic drugs for steatohepatitis. Both drugs have been shown to improve histology in small studies. [,16]

Silymarin

Milk Thistle, *silybum marianum,* has been used in both the treatment and prevention of liver diseases for over 2000 years. Silymarin, the active compound, consists of different flavonolignans including silibinin, isosilybin, silydianin, and silychristin. Among them, silibinin is the most biologically active compound, which makes up 50% of silymarin. The benefits of silymarin stem from its ability to function as a free radical scavenger and to stabilize plasma membranes. As free radical injury is often implicated in liver disease, silymarin has long been used in the treatment and prevention of hepatic disease.[,17,18]

Standardized extracts of milk thistle generally contain 70-80% silymarin, however, it is not regulated by the FDA. Pharmacokinetic studies estimate the bioavailability at 23-47%.[19] Peak plasma concentrations are reached within approximately 2 hours, and range between 200 and 1400ug/L. The elimination half life is approximately 6 hours in healthy volunteers.[,20] The majority of ingested silymarin undergoes enterohepatic circulation and 20-40% of the ingested dose is recovered in the bile as both glucuronide and sulfate conjugates. Bile levels of silymarin peak within 2 to 9 hours and excretion continues for 24 hours. A much smaller amount, 2-5%, is excreted in the urine while an even smaller amount is excreted unchanged in stool. Silymarin has poor bioavailability; however, studies have shown that combination with phosphphatidylcholine can increase biliary concentrations by as much as 3-11%.[,] Pharmacokinetic studies have also been performed in cirrhotic patients. Compared to healthy volunteers ingesting an equivalent dose of silymarin, the time to maximum concentration was increased in cirrhotic patients.[21]

Silymarin has been demonstrated in *in vitro* models to inhibit hepatic glucuronosyltransferases, most specifically UGT1A1[22,23] at micromolar concentrations. The clinical implication of this inhibitory profile on the potential for drug interactions is unclear, as is the potential for pharmacokinetic alteration in silibinin concentrations due to UGT inhibition of its own metabolism. Silymarin also appears to have modest inhibition of P glycoprotein (PgP), the gene product of MDR[24,25], although the clinical implication of PgP inhibitors remains unclear. Interestingly, Patel[26] found no changes in PgP mediated transport in the presence of silibinin, suggesting perhaps a differential effect of parent compound silymarin and its component isomers.

silymarin has been shown to have an excellent safety profile. Reported negative effects include nausea, diarrhea, dyspepsia, flatulence, abdominal bloating, pain or fullness as well as anorexia. Other reported adverse effects include headaches, skin reactions, asthenia, insomnia, arthralgia, impotence, and anaphylaxis.'[27] However, as stated by the Agency for Healthcare Research and Quality, in the majority of studies the incidence of adverse events was similar to placebo. Furthermore, given the lack of standardization of formulations for this agent it is possible that certain adverse events are a result of other ingredients included in the formulation.[28] In a review of clinical trials using silymarin versus placebo, no deaths or serious adverse events could be attributed to the study drug. In fact, more adverse events were reported in the placebo arm than the study arm.

Rationale for Silymarin in NASH

Through its antioxidant properties, silymarin may mitigate lipid peroxidation and the production of free radical injury. Studies evaluating the use of silymarin in this capacity have found it to be effective in scavenging hydroxyl radicals, preventing the release of TNF alfa, and restoring normal levels of superoxide dismutase, a precursor of glutathione.[29,30,31,32,33] Therefore, through these mechanisms, it is plausible that silymarin may have a therapeutic role in NASH.

Milk thistle has been studied in acute and chronic liver diseases such as alcohol, and viral hepatitis. Studies specifically evaluating the efficacy of silymarin for chronic HCV are scant, However, in 1992 Lirussi et al compared silymarin and ursodeoxycholic acid in patients with active cirrhosis who were predominantly HCV positive. No difference in blood chemistries with the use of either compound was detected.'[34] More recently, a silibinin compound was compared to placebo in 20 chronic active hepatitis patients. The silibinin compound was shown to reduce AST levels.[35] The potential benefits of silymarin in patients with chronic HCV may lie in the ability of this agent to mitigate the inflammatory and cytotoxic cascade of events initiated by viral replication.

Silymarin, compared to placebo, in alcoholic and non-alcoholic cirrhotic patients has been associated with decreased mortality attributable to liver disease.[36] However, in a more recent trial comparing silymarin to placebo in alcoholic patients with cirrhosis, no significant difference in mortality or biochemical parameters was found between the two groups.[37] One promising study evaluated the long-term effect of silymarin on hyperinsulinemia, exogenous insulin need, and MDA levels in people with insulin treated diabetes with alcoholic cirrhosis. Changes in these markers indicate that silymarin reduced lipid peroxidation and improved insulin resistance.[38]

Rationale for Subject Population

Patients with NASH will be enrolled and subsequently stratified by diabetes status. The majority of non-diabetic patients with NASH are expected to be overweight. Obesity and diabetes are the natural targets for study, given their ubiquity in the U.S. population and known association with NASH. Given the role of oxidative injury in the mechanism of NASH, silymarin's antioxidant properties would be a rationale for this approach to therapy.

The study population will exclude cirrhotic patients for several reasons. First, the histology of cirrhotics with suspected NASH often does not reflect the typical features of steatosis and steatohepatitis. Therefore, the diagnosis of NASH induced cirrhosis would be based on circumstantial evidence, such as the history of insulin resistance or obesity. Such assumptions of disease causation without histological confirmation would allow for potential misclassification

bias. Moreover, the primary endpoint of histological improvement could not be assessed in this population. Furthermore, people with cirrhosis demonstrate pharmacokinetics which differ from people without cirrhosis.

In the setting of a clinical trial, the safety of higher doses must be assessed, with a careful observation of the occurrence of clinical and laboratory adverse events. Doses of up to 1260 mg/day for up to 6 months were well tolerated in a small number of hepatitis C patients.[39] While silymarin appears to be extremely safe, the increase in dose could be associated with unexpected adverse effects. Additionally, there may be off targets of silibinin that could cause adverse drug reactions. Silymarin or silibinin have been implicated to have inhibitory effects on cytochrome (CYP) P450 enzymes in some,[,40,41,42] but not all *in vitro* studies. However, the inhibition profile of silibinin is theoretically relevant only for CYP 3A4 and 2C9, and then perhaps only in bile, which has drug concentration 100 times that seen in serum. Examination of enzyme inhibition in human clinical trials has been reassuring, with no evidence of altered metabolism of probe drugs specific to CYP 1A2, 2D6, 2E1 or 3A4[43,44,45] and of non-specific probes[46] when doses of 210-480 mg/day were administered. Silymarin does not appear to induce CYP 3A.[47]

In a trial designed to explore the efficacy of a therapy, it is important to control for potential confounding and interacting factors. Therefore, only patients who achieve and maintain a stable weight and lifestyle, to minimize the effect of such factors as change in weight or activity levels during the trial, will be enrolled. A weight range of +/- 10% change during the lead-in phase was chosen empirically; a weight change greater than this would be expected to be clinically significant and may affect (either improve or exacerbate) steatohepatitis, and thus function as a confounder. Among people with diabetes, the ideal patient population would be type 2 patients with insulin resistance who are not treated with any pharmacological agents. However, such stringent criteria would narrow the available subject population severely. As a result, patients with diabetes who are either diet controlled or who are receiving secretagogues with or without insulin will be enrolled. Therapy with secretagogues has not been suspected to mitigate the development of steatosis or the onset of steatohepatitis. Additionally, none of these medications is known to inhibit phase II metabolic processes and are thus less likely to influence silymarin disposition in humans. Similarly, although some secretagogues are metabolized by CYP isoforms, it is not anticipated that even the higher doses of silymarin used in this study will generate micromolar concentrations associated with CYP inhibition. In this phase II trial, the placebo control will allow the research team to discern the treatment effect attributable to silymarin, and thus support the study's validity as a pivotal trial and advancement to phase III studies.

Dose Selection

As stated in the Phase I protocol, decisions regarding dose selection for the Phase II trial are primarily based upon consideration of safety and PK relationships observed for the sum of parent (i.e. "Free") silymarin flavonolignan concentrations (i.e. ∑ Free silymarin). However, an unexpected finding from the Phase I study was that ∑ Free silymarin accounted for only 1.7% to 7.2% of the Total concentrations of silymarin flavonolignans found in the blood for NAFLD subjects receiving 280 to 560 mg silymarin three times daily (Appendix 1, Summary Table 4). The remaining flavonolignan species that contribute to Total concentrations consist of glucuronide and sulfate conjugates (i.e. metabolites) of the parent flavonolignans. Since parent ("Free") silymarin flavonolignans undergo extensive first-pass phase 2 metabolism to glucuronide and sulfate conjugates in the GI tract and in the liver, use of ∑ Free silymarin concentrations to assess drug exposures will be imprecise since it does not represent the total

amount of drug entering the body. In addition, since conjugate concentrations in blood are approximately 11.5-times greater than “Free” concentrations, a small reduction in the extent of silymarin’s metabolism to conjugates would be expected to greatly influence “Free” blood concentrations without changing “Total” blood concentrations or ∑ Total silymarin exposures. In addition, small changes in the extent of conjugation are not easily detected in “Total” concentrations because of the high %CV in these measures.

Therefore, “Total” flavonolignan concentration, which represents all species of a flavonolignan in blood, is the best measure of the amount of a flavonolignan entering the body following an oral dose. Because any or all of the silymarin flavonolignans may contribute partially or fully to the pharmacological activity of silymarin, ∑ silymarin concentration, which represents the sum of the concentrations of all six major silymarin flavonolignans, is the best measure of silymarin exposure for assessment of safety and efficacy endpoints. Given these considerations, mean ∑ Total silymarin AUC was chosen for the assessment of dose-exposure proportionality in the Phase I trial and provides the basis for the selection of 420 mg and 700 mg doses administered three times daily in this Phase II study.

Rationale for Dose Selection

High Dose: 700 mg Silymarin three times daily.

A dose-proportional 2-fold increase in [∑] Total silymarin exposures (i.e. AUC) was observed between 280 mg and 560 mg NAFLD dose groups (Appendix 1, Summary Table 6). While not assessed in NAFLD subjects, dose-proportional increases in ∑ Total silymarin AUCs were observed up to a dose of 700 mg in HCV subjects (Appendix 1, Summary Table 5) suggesting that ∑ Total silymarin exposures are predictable and reliable up to 700 mg. In addition, the ∑ Total silymarin exposures observed with doses of 280 and 560 mg in NAFLD subjects were only ~60% of the ∑ Total silymarin exposures attained with these same doses in HCV subjects (Appendix 1, Summary Table 7). Therefore, it is expected that for NAFLD patients ∑ Total silymarin exposures at a dose of 700 mg will be substantially less than the ∑ Total silymarin exposures obtained with 700 mg in HCV patients.

Since AEs were not observed with the highest exposures attained with the 700 mg dose in HCV subjects, a silymarin dose of 700 mg administered three times daily can be expected to provide reliable exposures and to be safe and well tolerated in this NASH SyNCH Phase II trial. Also, a dose of 1680 mg daily (560 mg administered thrice daily) appeared to be safe and well tolerated in patients with NAFLD (Appendix 2). Two of 12 subjects randomized to Legalon® reported a mild adverse event, one unrelated and one unlikely related to study drug. Both adverse events reported with a dose of 560mg administered three times daily. In addition, this dose burden (5 pills three times daily) is the largest expected to be possible and still have reasonable patient compliance.

Non-Linear Pharmacokinetics of ∑ Free silymarin plasma concentrations

“Free” parent flavonolignan concentrations can be expected to have influences on the efficacy and safety of silymarin that must be considered independent from those associated with “Total” silymarin flavonolignan concentrations. Non-linear pharmacokinetics was observed for [∑] Free silymarin exposures in NAFLD subjects since a 2-fold increase in dose from 280 mg to 560 mg resulted in a 9-fold increase in the ∑ Free silymarin AUC (see Appendix 1, Summary Table 2). ∑ Free silymarin peak concentrations increased ~4-fold and ranged from 0.50 to 1.77 ug/ml at a dose of 560 mg in NAFLD subjects (Appendix 1, Table 15). At a dose of 560 mg, mean ∑ Free silymarin exposures for NAFLD patients (4387 ng*hr/ml) were 4-fold greater than those for HCV patients (1025 ng*hr/ml) and higher than those attained with a dose of 700 mg in HCV patients

(3361 ng*hr/ml) where the ∑ Free silymarin_peak concentrations ranged from 0.42 to 2.71 ug/ml. These data are reported in Appendix 1, Table 15 and Summary Tables 1 and 2. Therefore, a dose of 700 mg will likely result in ∑ Free silymarin exposures for NAFLD subjects that will be higher than those observed with this dose in HCV subjects.

Since non-linear pharmacokinetics was observed at 560 mg, the highest dose evaluated in the Phase I study with NAFLD subjects, it is possible that there will be substantially higher ∑ Free silymarin exposures and peak concentrations in this Phase II trial with a 700 mg dose than what was attained in the Phase I study for either HCV or NAFLD subjects. This possibility raises potential concerns for safety and tolerability since Flaig et al. (2007)[48] found free silymarin flavonolignan concentrations above 10-12 ug/ml were associated with reversible grade 1 to grade 3 elevations in liver transaminase and unconjugated bilirubin concentrations in a Phase I study with a different formulation of two silymarin flavonoligans in patients with prostate cancer.

To balance the need to achieve the highest exposures possible and thus have the greatest chance of achieving efficacy for NASH patients, with the concern for patient safety, 700 mg three times daily was selected as the highest dose in NASH patients but will employ an early pharmacokinetic monitoring plan for ∑ Free silymarin steady-state plasma concentrations (see Section 3.4.4) in addition to our safety assessments. Comparison of ∑ Free silymarin plasma concentrations achieved after 2 weeks of chronic dosing with the peak plasma concentrations and associated adverse events reported by Flaig et al will make it possible to anticipate the need for greater vigilance for elevations in liver enzymes and bilirubin with the continuous chronic administration of 700 mg silymarin. Dose reductions or discontinuations for elevated liver enzymes and bilirubin are included in the routine safety procedures (see section 3.4.1).

Low Dose: 420 mg Silymarin three times daily
Since dose-exposure proportionality was observed in ∑ Total silymarin exposures over the dose range of 280 to 560 mg silymarin in NAFLD subjects, and 140 to 700 mg silymarin in HCV subjects, a dose of 420 mg silymarin will be used as the lower dose in this Phase II trial as it represents a dose predicted from Phase I data that will result in 40% lower exposures which might be associated with clinically significant differences in pharmacodynamic endpoints when compared to those observed at a dose of 700 mg.

2.0 Study Objectives and Endpoints

2.1 Primary Objectives:

1. To assess the safety and adverse event profile of silymarin over a range of doses compared to placebo
2. To assess the efficacy of silymarin as defined by an improvement in NAS by at least 2 points.

2.2 Secondary Objectives:

1. To compare NAS before and after therapy
2. To characterize changes in ALT during silymarin therapy
3. To compare HOMAr before and after silymarin therapy
4. To explore relationships between silymarin therapy and serum biomarkers (oxidative stress, apoptosis, and fibrogenesis)

5. To characterize the population pharmacokinetics and pharmacodynamics of silymarin isomers including, silibinin A and B, isosilibinin A and B, silicristin, and silidianin following administration of silymarin to subjects with NASH.

2.3 Endpoints

Efficacy: The primary outcome variable (endpoint) for efficacy is improvement by at least 2 points in histology (NAS).

Safety: The primary outcome variable for safety is the occurrence of a dose-limiting toxicity during the 48-50 week treatment period. Adverse events (AE) will be categorized by severity.

Adherence: Will be measured using a summary of missed dose information obtained from patient diaries and dose counts and will be analyzed as both an outcome and as an explanatory variable in exploratory analyses of pharmacokinetic exposure and response.

Biomarkers: The following relationships will be explored: dose and change in biomarkers, changes in biomarkers and treatment success, changes in biomarkers and toxicity.

Secondary

- Improvement by at least 1 point in histology (NAS) after therapy
- ALT or AST ≤ 45 IU/L (approximate normal range) or with greater than 50% reduction in ALT or AST to a value less than 65 IU/L (approximately 1.5 times the upper limit of normal) after therapy
- Insulin resistance measured by HOMAr.

3.0 Study Design

3.1 Study summary

This will be a phase II, multicenter, randomized, double-masked, placebo-controlled study to assess the safety and efficacy of a standardized silymarin preparation (Legalon®) for treating patients with NASH, as defined by a NAS score of at least 4 on a baseline biopsy. Participating centers and Principal Investigators in the study are shown in Table 1. A total of 120 subjects will be enrolled at the 4 clinical centers. All subjects will be at least 18 years old, have serum AST or ALT > 40 IU/L and demonstrated fatty liver. After careful evaluation, all eligible subjects will be entered into a 28-day lead-in phase in which they will undergo counseling with respect to diet, exercise, and alcohol consumption. Participants successfully completing the lead-in phase will be randomized to treatment with one of two dosages of Legalon® or a placebo. Legalon® will be administered orally thrice daily for 48-50 weeks. Subjects will be evaluated regularly for adverse events and for monitoring efficacy through blood sampling. After completing therapy, subjects will be monitored for an additional 12 weeks to measure any sustained effects of study medication and to continue to monitor for resolution of any adverse events.

Table 1
Participating Centers

Institution	Location	Principal Investigator	Type of Center
Beth-Israel Deaconess Medical Center	Boston, MA	Nezam Afdhal, MD	Clinical Center

Thomas Jefferson University	Philadelphia, PA	Victor Navarro, MD	Clinical Center
University of North Carolina	Chapel Hill, NC	Michael W. Fried, MD	Clinical Center
University of Pennsylvania	Philadelphia, PA	K. Rajender Reddy, MD	Clinical Center
University of Pittsburgh	Pittsburgh, PA	Steven H. Belle, PhD	Data Coordinating Center

A schematic of the study design is shown below. The required study visits and evaluations that will be performed at various time points are shown in subsequent tables.

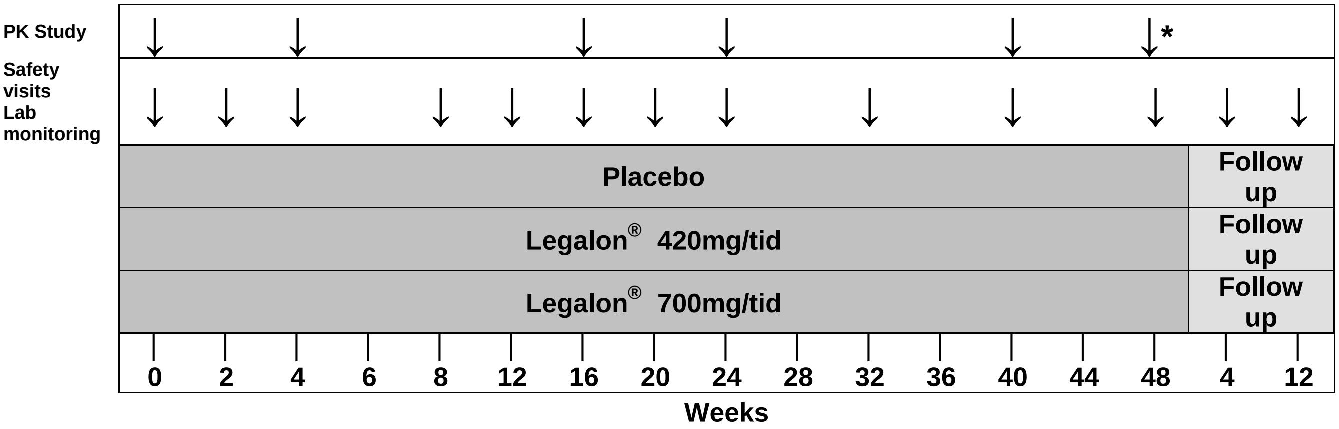

***** Last dose (day before follow-up biopsy) & day of follow-up biopsy

3.2 Study Population

Participants will be at least 18 years old, have serum AST or ALT > 40 IU/L, and demonstrated fatty liver.

3.3 Entry Criteria

To be eligible for randomization, patients must meet all of the inclusion criteria and none of the exclusion criteria below:

Inclusion Criteria

- Age at least 18 years at the start of the lead-in period
- AST or ALT greater than 40 IU/L within one year of the start of the lead-in phase and at least once during the lead-in phase
- Successfully complete a 28-day lead-in phase. Successful completion of the lead-in phase will require the participant's BMI at the end of the lead-in period to be within +/- 10% of their BMI obtained at the initial lead-in visit, as well as adherence to the alcohol consumption guidelines.
- Have a liver biopsy performed within 18 days of completing the lead in phase demonstrating features consistent with NASH without cirrhosis; NAS score of at least 4.
- Negative urine pregnancy test (for women of childbearing potential) documented within the 24-hour period prior to the first dose of study medication. Females of childbearing

potential must be using two reliable forms of effective contraception during the study (while on study drug and during follow-up)

- Demonstrated fatty liver on an imaging modality, either ultrasound, CT, or MRI, within 1 year of the start of the lead-in phase
- Participant agrees to discontinue use of other antioxidants such as vitamin E, vitamin C, glutathione, alpha-tocopherol, or non-prescribed complementary alternative medications (including dietary supplements, megadose vitamins, herbal preparations, and special teas) from the start of the lead-in phase through completion of the follow-up phase. A multivitamin at standard doses will be allowed.
- Participant agrees to discontinue use of silymarin or other milk thistle preparations, other than study medication, from the start of the lead-in phase through completion of the follow-up phase

Exclusion Criteria

- BMI $\geq$ 45 kg/m^2 between the first lead-in visit and randomization
- Type 2 diabetes treated with oral agents other than the secretagogues or metformin; these include, thiazolidinediones, alpha-glucosidase inhibitors, exenatide, pramlintide between the start of the lead-in phase and randomization. Januvia (sitagliptin) is allowed.
- Evidence of poorly-controlled diabetes (Hba1c > 8%) between the first lead-in visit and randomization
- Known allergy/sensitivity to milk thistle or its preparations
- Use of drugs associated with a clinical or histological picture consistent with fatty liver disease or NASH for more than 12 consecutive weeks in the 1 year prior to start of the lead-in phase; these include amiodarone, tamoxifen, methotrexate, glucocorticoids, anabolic steroids, tetracyclines, estrogens at doses greater than those used for hormone replacement, valproate/valproic acid
- For patients using antihyperlipidemic agents or accepted anti-diabetic agents, any change of agent or dose from the start of the lead-in phase through randomization
- Use of warfarin, metronidazole, or acetaminophen (greater than 2 grams per day) from the start of the lead-in phase through randomization
- Lactose intolerance defined as patient reported inability to tolerate milk products
- History of other chronic liver disease, including metabolic diseases, documented by appropriate test(s)
- Previous liver biopsy that demonstrated presence of cirrhosis
- Radiologic imaging consistent with cirrhosis and portal hypertension
- Clinical or histological evidence of cirrhosis or, in the opinion of the investigator, the inability to safely obtain a liver biopsy due to technical reasons, such as body habitus.
- Evidence of decompensated liver disease defined as any of the following: serum albumin <3.2 g/dl, total bilirubin > 1.5 mg/dl, or PT/INR > 1.3 times normal from the start of the lead-in phase through randomization, or history or presence of ascites or encephalopathy, or bleeding from esophageal varices
- Platelet count < 130,000/mm^3 at any time from the first lead-in visit through randomization
- Serum creatinine of 2.0 mg/dL or greater or CrCl ≤ 60cc/min, or on dialysis, at any time from the first lead-in visit through randomization. The creatinine clearance (CrCl) will be calculated according to Cockcroft-Gault.
- Average alcohol consumption of more than one drink or equivalent (>12 grams) per day and no more than two (2) drinks on any one day over the 30 days prior to the start of the

lead-in phase. Patients who met either criterion more than 30 days prior to the start of the lead-in phase must have consumed a monthly average of 12 grams or less per day of alcohol for at least six months prior to the start of the lead-in phase

- Evidence of drug abuse in the year prior to the start of the lead-in phase or prior to randomization
- History of immunologically mediated disease (e.g., inflammatory bowel disease, idiopathic thrombocytopenic purpura, lupus erythematosis, autoimmune hemolytic anemia, severe psoriasis, rheumatoid arthritis) that could affect the assessment of biomarkers of inflammation
- History of solid organ or bone marrow transplantation
- History of thyroid disease poorly controlled on prescribed medications
- Use of oral steroids for more than 14 days within 30 days of the start of the lead-in phase or prior to randomization
- Primary hepatic malignancy
- Secondary hepatic malignancy (metastatic disease) or extrahepatic malignancy
- Women with ongoing pregnancy or breast feeding, or contemplating pregnancy
- History of bariatric surgery, or undergoing evaluation for bariatric surgery.
- Participation in a research drug trial, exclusive of the SyNCH Phase I trial, within 30 days of the first lead-in visit
- History or other evidence of severe illness or any other conditions that would make the patient, in the opinion of the investigator, unsuitable for the study (such as poorly controlled psychiatric disease, coronary artery disease, or active gastrointestinal conditions that might interfere with drug absorption)
- Inability or unwillingness to give informed consent or abide by the study protocol

3.4 Study Treatment

Participants will be randomized to treatment with either Legalon® 420 mg or 700 mg, or placebo administered thrice daily. The treatment period will be for 48-50 weeks and subjects will be followed off treatment for an additional 12 weeks. An IND (#74887) has been filed for the use of Legalon® in this study and a dossier reflecting additional preclinical and clinical information about this agent accompanies this protocol (Appendix 3).

Each patient will receive 5 capsules as outlined in Table 2 for each orally administered dose that will consist of Legalon® and/or placebo in the appropriate capsule quantities to standardize pill burden, allow for an estimate of compliance, and to minimize the potential for variable absorption due to differential solubility in the gastrointestinal tract.

Legalon® (Rottapharm/Madaus) is a milk thistle fruit extract standardized to 140 mg silymarin per capsule (53% as total silybins). A Legalon® 140 capsule contains 180 mg dried extract of milk thistle fruits, or 140 mg silymarin which is the presumed active ingredients, or 108 mg silymarin quantitated as silybin by HPLC. The total weight of the capsule is 429 mg and includes: other milk thistle components that are not chemically well defined; components of the extraction process; excipients; and the capsule shell. The 108 mg of silymarin in Legalon® 140 capsules consists of the following potentially active ingredients: silybins A + B (~50 mg), silychristin (~20 mg), silydianin (~15mg), and isosilybins A + B (~15 mg).

Table 2
Dosing Strategy for Standardizing Pill Burden

Legalon® Dose	# Legalon® Caps	# Placebo Caps	Total # of Capsules per Dose
Placebo	0	5	5

420 mg	3	2	5
700 mg	5	0	5

3.4.1 Dose Adjustment Guidelines
Specific dose adjustment guidelines are provided to investigators for adverse effects considered to be possibly related to silymarin, including laboratory abnormalities, adverse events, and vital signs changes, investigators should utilize the "General Dose Reduction Guidelines" (Section 3.4.2) below. When practical, abnormal laboratory results should be confirmed as soon as possible following notification of the investigator. If appropriate, downward adjustments in one level increment (see below) should be considered.

3.4.2 General Dose Reduction Guidelines
Dose reduction for adverse events will be based upon severity ratings in accordance with Common Toxicity Criteria for Adverse Events (CTCAE) (Appendix 4). For grade 1 toxicity, no dose reduction is needed. For toxicity grade 2 that persists over two consecutive safety visits and is not responsive to adjunctive symptomatic management, a level 1 dose decrease of study medication may occur at the discretion of the investigator. For toxicity grade 3 not responsive to adjunctive measures, a level 1 dose decrease of study medication is required. Subjects with grade 3 toxicity should be followed closely, possibly at more frequent intervals or via telephone contact to monitor in between regularly scheduled safety visits. If symptoms improve, return to previous dosing is allowed at the discretion of the investigator. For grade 4 toxicities, treatment will be discontinued. It should be noted that certain toxicities carry different levels of significance for each patient and therefore, the investigator should use these as guidelines only, within the context of clinical judgment.

3.4.3 Dose-Specific Reduction Guidelines

Dose Reduction Guideline – Silymarin 700 mg dose level

AE Grade	Reduction?	Discontinue?	Silymarin	Placebo	Pill Burden
Mild	No	No	5	0	5
Moderate*	Yes	No	4	1	5
Severe**	Yes	Temp	3	2	5
Life Threatening	No	Yes	0	0	0

Dose Reduction Guideline – Silymarin 420 mg dose level

AE Grade	Reduction?	Discontinue?	Silymarin	Placebo	Pill Burden
Mild	No	No	3	2	5
Moderate*	Yes	No	2	3	5
Severe**	Yes	Temp	1	4	5
Life Threatening	No	Yes	0	0	0

**Severe AE: Discontinuation for 1 week. If SAE improves or resolves, restart at indicated dose level at the investigator’s discretion. The dose level may be increased to original dose level at the discretion of the investigator with continued close monitoring.
*Moderate AE: Patient can resume original dose level if AE improves or resolves. NOTE: If the subject experiences a drug related AE and two dose reductions are not tolerated, the subject will stop the drug

3.4.4 Safety measurements

Laboratory evaluations
Participants will undergo laboratory tests according to the time and event schedule to monitor for medication toxicities.

Pharmacokinetic Safety Monitoring
Blood samples will be collected and processed at each site and then shipped to the NIDDK repository. Aliquots needed for PK testing will be shipped to the central analytical lab at UNC. .Treatment week 2 samples from the first 6 patients receiving the highest dose (700 mg three times per day) will be used for bioanalysis to determine the range of ∑ Free silymarin concentrations. Depending on the results, the determination of testing blood samples obtained from subsequent subjects will be made.

Adverse event monitoring
Participants will be queried by study personnel at each study visit about the incidence of adverse events. These will be documented in the study record and classified according to duration, severity, relationship to study medication, and action with study medication. Serious adverse events will be reported to appropriate regulatory authorities, in addition to the DSMB as below, in a timely fashion.

In the event that an investigator considers an adverse event to be related to study medication, the investigator may submit an unmask request to the clinical center investigational pharmacist to unmask the study drug dose, per the study specific unmasking procedure.

Pregnancy
Women of child-bearing potential will have a urine pregnancy test performed within 24 hours of the first administration of study drugs, monthly for the duration of therapy, and at 1 month following completion of therapy.

3.5 Premature discontinuation

Treatment may be discontinued prematurely for the following reasons:

1. Participant requests termination of the study medication
2. Participant is intolerant of the study medication despite predetermined dose reductions and side effect management strategies
3. Safety concerns at the discretion of the investigator. These concerns will be documented in the source documents

Participants who discontinue treatment prematurely for the above reasons will be encouraged to remain in the post-treatment follow-up period for an additional 12 weeks, unless they withdraw consent. For those discontinued prematurely, blood samples will be obtained as listed for the week 48 end-of-treatment visit.

Patients who drop out after 6 months of treatment will be asked to undergo the liver biopsy. Those who drop out after fewer than 6 months of treatment or fail to undergo the follow up liver biopsy will be asked to undergo laboratory studies according to the follow up protocol.

4.0 Study visits (Appendix 5)

At each visit, patients will be advised to limit alcohol intake to no more than one drink or equivalent (>12 grams) per day and no more than 2 drinks on any one occasion, and to record alcohol intake into a diary. Diet modification is usually accompanied by a recommendation to exercise 30-45 minutes a day, 3-5 times a week[49] Patients will be counseled to avoid non-prescribed medicines or complementary alternative medicines, including milk thistle preparations. Patients will be instructed to complete a diary through the lead in and dosing periods. The diary used during the lead in period will help assess patient compliance to diet, exercise, and alcohol regimen. The diary used during the treatment period will help assess study medication dosing and alcohol use. Patients will be instructed to record adverse events and concomitant medications. This will be reviewed at all visits.

4.1 Lead-In Phase

The lead-in phase will be comprised of 2 visits, 28 days apart. Overweight and obesity are now defined by using Body Mass Index (BMI). BMI is defined as weight (in kilograms) divided by height squared (in square meters). Each patient will be counseled to achieve and maintain a target weight/BMI. A target of 10% of baseline weight is often used as an initial goal of weight loss according to the NHLBI-NIDDK. During each visit of the lead-in phase, patients will have blood drawn for liver tests, the BMI determined, and undergo dietary counseling by a member of the study staff. Patients will be counseled to follow a healthy diet and lifestyle as recommended by the U.S. Department of Health and Human Services and the U.S. Department of Agriculture. Dietary recommendations include a decrease in saturated fats as well as total fats to <30% of total calories and macronutrient distribution of 45 to 55% carbohydrate, 25 to 35% fat and 15 to 20% protein. Patients will be provided with dietary counseling to maintain glycemic control as well as to maintain a target weight/BMI that reflects no more than a +/- 10% change of body weight, as measured during the first lead-in visit. Guidelines for diet and exercise counseling are appended (Appendix 6).

4.2 Lead-In Visits

Visit 1

Subjects will give a complete medical history and, have a physical examination. Patients will be advised to limit alcohol intake to no more than one drink or equivalent (>12 grams) per day and no more than 2 drinks on any one day, and to record alcohol intake into a diary. Moderate levels of exercise will be recommended for 30-45 minutes per day, 3-5 times a week.

All patients will have blood drawn for liver enzymes, blood chemistries, and a complete blood count including platelet count. The BMI will be determined, and they will undergo dietary counseling in a 30-minute session by a registered dietitian. Each patient will be counseled to achieve and maintain a target weight/BMI that reflects no more than a +/- 10% change in BMI between the 2 lead-in visits. Dietary recommendations include a decrease in saturated fats as well as total fats to <30% of total calories and macronutrient distribution of 45 to 55% carbohydrate, 25 to 35% fat and 15 to 20% protein.

Subjects will be instructed to complete a diary to record alcohol consumption, concomitant medications, and adverse events. This will be reviewed at all visits.

Visit 2 (Day 28)

Subjects will return for the final lead-in visit to evaluate whether they can proceed to the treatment period. At this visit, a physical examination (including weight and vital signs), BMI and any changes in medical history as compared to the first lead-in visit will be recorded. Blood tests will be done for complete blood count including platelet count, chemistry, liver enzymes, and a routine urinalysis. Blood will be collected for future studies. Patients with at least one liver enzyme greater than 40 IU/L (AST or ALT) will have a liver biopsy performed ideally within 14 days, but allowable up to 18 days, of completion of the lead-in phase. The liver biopsy results must be obtained prior to moving on to the treatment phase.

Repeat Lead-in Procedure

Eligible patients must be randomized within 28 days of completion of the lead-in phase. Ideally, a liver biopsy will be performed within 14 days of completion of the lead in phase. The biopsy may be performed up to 18 days following the completion of the lead in phase, provided the patient still meets the +/- 10% change in BMI from the start of the lead-in phase and has adhered to the alcohol consumption guidelines, as determined at the time the biopsy is performed. If the biopsy is not performed within 18 days of completing the lead-in period, the 28 day lead-in period must be repeated. If the biopsy is performed within 18 days of completing of the lead-in period but the patient is not randomized within 28 days of completing the lead-in period, the patient may still be randomized up to 42 days following completion of the lead-in period. If the patient is randomized between 29 and 42 days after completing the lead-in period, the laboratory tests (liver enzymes, blood chemistries, and blood count) must be repeated, and the patient must still meet the +/- 10% change in BMI from the start of the lead-in phase and has adhered to the alcohol consumption guidelines.

4.2 Baseline/Randomization Procedures

Liver biopsy

Percutaneous liver biopsy will be performed within 28 days of randomization, during the 28-day period following completion of the lead in phase. The liver biopsy will be performed according to each sites' standard procedures; however, an adequate specimen of at least 1.5 cm in length is recommended. The biopsy core will be processed for histological analysis. The pathologist at each clinical site, or at the central pathology site as described below, will score the liver biopsy according to a grading scheme as described by Brunt et. al. and Promrat et.al., and as modified by the Nonalcoholic Steatohepatitis Clinical Research Network (NSCRN) recommendation. In the NSCRN grading scheme, five features were independently associated with the diagnosis of NASH: steatosis, ballooning, lobular inflammation, fibrosis, and absence of lipogranulomas[12]. A NAS score of at least 4 detected by the local or central pathologist, as determined by each investigator, will be required for entry (Appendix 7).

Four slides from each patient's liver biopsy will be sent to a central pathology site at the Beth Israel Deaconess Medical Center, Boston, MA. The central pathology site will stain the slides (hematoxylin/eosin, trichrome) and the study pathologist will evaluate each biopsy at the end of the study without knowing which slides belong to which patients and assign a NAS. Pre- and post-treatment biopsy NAS will be determined for the primary efficacy analysis. Patients who meet all inclusion and no exclusion criteria at the end of the lead-in period and have liver biopsy findings consistent with NASH (NAS $\geq$ 4) will be randomized. A baseline (randomization) visit will take place within 28 days of successful completion of the lead-in period and following the liver biopsy. During this 28-day period, the inclusion and exclusion criteria will be verified. In addition, the following procedures will take place.

- A history and physical examination will be conducted
- Baseline laboratory studies including liver tests, complete blood count, serum electrolytes, BUN and creatinine, fasting glucose and insulin, and serum silymarin will be obtained
- Fasting serum and urine will be collected for storage and biomarker studies (oxidative stress, apoptosis, and fibrosis)
- Urinalysis
- HOMAr, determined by the formula:

$$\frac{\text{Glucose mg/dL x 0.05551) x Insulin mcUI/mL}}{22.5}$$

- Urine pregnancy test for women of childbearing potential
- Subjects will be advised on the following:
    - Maintain a diet and exercise program to support maintenance of weight with calorie requirements based upon usual food intake as determined by food intake diaries and diet histories
    - To maintain a lifestyle/ diet/ exercise program
    - Not to consume alcohol in excess of one drink (12 grams) per day and no more than 2 drinks on any one day
    - Not to consume any non-prescribed medicines, naturopathic remedies, or vitamins
- Contraceptive plans for women of childbearing potential
- Quality of life assessment (SF-36,), depression (CESD), symptom assessment (CLDQ), and Adherence Self Efficacy (ASE)
- Randomization, upon review of eligibility data

4.3 Treatment Phase

Study visit procedures

Study visits will occur at weeks 2, 4, 8, 12, 16, 20, 24, 32, 40, and 48 while the subjects are in the treatment phase of the study. Phone calls will be made to the participants at weeks 6, 28, 36 and 44 to monitor safety, compliance with study medication, and concurrent medications. There is an acceptable window +/- 1 day for visits at week 2, and 4, and a window of +/- 3 days for all other visits.

On the evening prior to the clinic visit at Week 2, study coordinators will instruct subjects by phone call to withhold their morning dose until they come to the clinic. This clinic visit will be scheduled around a subject's silymarin dosing such that prior to the end of the clinic visit, coordinators will witness the subject's ingestion of their dose and will then have a blood sample drawn between 30 minutes to 1 hour after dosing for the estimation of silymarin steady-state peak and trough plasma concentrations.

The following procedures will be performed at the study visits:

Interim Medical History	
Symptom-directed Physical Exam	Vital signs and weight at each visit; physical exam every 3 months or as needed
Adverse Event Assessment	Each visit

Concurrent Medication Assessment	Each visit
CBC/Differential	Each visit
Chemistry	Each visit; ALT, AST, AlkPO4, total bilirubin, direct bilirubin, albumin, BUN, creatinine, glucose.<br>Week 12, 24, and 48 only: fasting triglycerides, glucose, insulin
Pregnancy Test	Urine, each visit
TSH	Weeks 24 and 48
PTor INR	Weeks 24 and 48
Silymarin level	Weeks 2, 4, 12, 16, 24, 32, 40, 48
EKG	Week 12
Sample Storage	20ml serum, 20ml whole blood, 10ml plasma, 24ml urine each visit from week 2 to week 24 and then week 48 (fasting at weeks 12, 24, and 48)
Pharmacokinetic Sample	Each visit according to assigned schedule or random sample
Liver biopsy and CBC, ALT, AST, HOMAr	Week 48 (or end of treatment)
Adverse Event Assessment for Mental Health	Each visit
Questionnaires	Weeks 4, 12, 24, 48: SF-36, CLDQ, CES-D, ASE
Adherence Monitoring and Counseling	Each visit<br>Monitoring by dose counts and review of diary entries

Compliance Monitoring

Compliance will be assessed at the clinical sites by dose count and review of diary entries. In addition, weight/BMI determinations will be made during the study; because of the rigors associated with diet, exercise, and lifestyle consumption restrictions.

4.3.1 Pharmacokinetic study

Specific goals of the study pharmacokinetic analysis will include 1) confirming estimates of PK parameters obtained during phase I, in which only a small number of NAFLD patients were enrolled, 2) a comparison with the HCV population, 3) identifying the sources and magnitude of intersubject pharmacokinetic variability on the basis of covariates, 4) observing changes in metabolite kinetics over time, and 5) potentially linking safety and outcome measures to pharmacokinetic profiles. The specific analyses are outlined below in section 6.4.

Subjects

The PK sub-study will provide samples according to the limited sampling strategy described below and will be used to develop a more robust population PK model and will also provide for an evaluation of the extent of intra-subject variability influencing covariates under investigation.
All patients enrolled in the main study will be eligible to participate in the PK sub-study until the PK sub-study groups are filled. Of the 120 subjects enrolled in the main study, the first 30 who provide consent for the PK sub-study will be enrolled in the sub-study, 10 subjects per dose group.

Replacement dropouts

The PK sub-study is using this limited subset of subjects to "predict" PK of the study population as a whole. For this reason participants who drop out the PK study will be replaced to successfully fill each dose group.

Sampling Design

A limited sampling strategy will be employed in which a total of 54-60 ml (9-10 samples x 6 ml/sample) of blood will be drawn per patient for quantifying silymarin isomers. Sampling time windows have been selected to optimize PK information from a sparse sampling schedule based on our preliminary model The 10 subjects in each dose group will be randomly assigned by the Data Coordinating Center to either Schedule 1 or 2 which will require additional PK sampling at 3 or 4 of the clinic study visits; 5 of these subjects will be sampled on Weeks 0, 24, and 48, while the other 5 subjects will be sampled on Weeks 4, 16, 40. Actual sample collection times will be recorded on the case report forms and be utilized in the subsequent analyses.

Sequential blood samples will be obtained from each subject on the same day according to the sampling schedule into which the subject is assigned.

Phase II NASH Trial

Schedule 1:	First Dose:	0-1 hours, 1-3 hours, 3-6 hours
	Week 24:	0 hour (trough), 1-2 hours, 2-4 hours
	Last dose (Day before follow-up Biopsy):	0 hour (trough), 4-6 hours
	Day of follow-up biopsy:	1 to 2 samples between 18-24 hours following last draw above*

Schedule 2:	Week 4:	0 hour (trough), 1-2 hours, 2-4 hours
	Week 16:	0 hour (trough), 1-2 hours, 2-4 hours
	Week 40:	0 hour (trough), 1-2 hours, 2-4 hours

At the time of a scheduled study visit, which can be any time during the day, 6 ml blood samples will be obtained from all other study subjects not participating in the PK sub-study. Study coordinators will record the time of the most recent dose from subject report and record the time of sample collection. The inclusion of this sampling scheme will be used to further develop a robust population PK model and will provide for an evaluation of the extent of inter-subject variability influencing the covariates under investigation.

Blood will be collected for silymarin (Legalon®) and silymarin metabolite concentration (silibinin A and B, isosilibinin A and B, silicristin, and silidianin) at baseline and treatment weeks 2, 4, 12, 16, 24, 32, 40, and 48. Special attention will be paid to recording in source documents accurate sample collection times and timing of previous silymarin dose. Serum concentrations of free and total silymarin isomers will be quantified using LC/MS. Determination of the total fraction will be accomplished after hydrolysis with glucuronidase/arylsulfatase. The extent of phase 2 metabolism will be estimated from the quantitation of the amount of conjugated silymarin

* Time of visit relative to administration of last dose. If close to 18 hrs at time of visit then also collect a 22-24 hrs timepoint depending on length of stay. If 22-24 hrs at time of visit then only 1 draw is needed. Prefer two draws in this window in order to determine the terminal half-life of the drug. The draws need to be at least 2 hrs apart.

isomers that will be calculated as the difference been the total and free fractions for each isomer.

Blood
Blood will be collected from each subject for plasma silymarin concentration determination at the times indicated in the schedule of events tables. Blood samples will be collected with plasma samples transferred to labeled polypropylene tubes, sealed, and stored immediately at -20ºC in an upright position until shipped on dry ice.

Urine
Two 10 ml aliquots (20 ml total) of urine from each collection interval will be taken and frozen for determination of silymarin concentrations.

Analysis of silymarin concentrations in plasma and urine will be performed using validated analytical methods. All biological samples will be shipped to and stored at the NIDDK repository and then shipped to the testing laboratory for analysis at the discretion of the SyNCH Investigators

The label for each sample will identify the study number and collection date and time.

4.4 Follow-up period

Within two weeks of completing the 48 week treatment period, liver biopsy will be performed, as previously described, while the patient continues on silymarin. Therefore, patients may receive as many as 50 weeks of treatment. Follow up laboratory studies including ALT, AST and HOMAr will be obtained at the time of the liver biopsy. Also, blood will be collected for future studies.

Subjects will be followed for an additional 12 weeks after end of treatment. They will return for post-treatment visits at follow-up weeks 4 and 12. During these visits, participants will be evaluated for resolution of adverse events and laboratory studies will be drawn to measure efficacy. The following procedures will be performed:

Interim Medical History	
Symptom-directed Physical Exam	Vital signs,weight, and physical exam as needed
Adverse Event Assessment	Each visit
Concurrent Medication Assessment	Each visit
CBC/Differential	Each visit
Chemistry	Each visit: ALT, AST, AlkPO4, total bilirubin, direct bilirubin, albumin, uric acid, BUN, creatinine, glucose<br>HOMAr at week 12 only
Pregnancy Test	Urine, Post-treatment week 4 only
Sample Storage	20ml serum, 20ml whole blood, 10ml plasma, 24ml urine each visit
Questionnaires	Week 4: SF-36, CLDQ, CES-D, ASE

4.5 Unscheduled visits: Participants who have changes in severity or initiation of new symptoms, or who need to have laboratory tests repeated, will be asked to return to the study site for unscheduled evaluations.

5.0 Adherence Measures

It is recognized that there is a significant pill burden (15 capsules daily) for this study. Therefore, an effort will be made to measure adherence to the medication regimen in order to assist with the efficacy analysis and provide information that will be helpful if a phase III study is planned.

Adherence Measures

Procedures

Patient Education and Adherence Program

The adherence program for the study will consist of three central components: (a) informational-exchange, (b) skills development, and (c) social support enlistment. One of the tools utilized in this study will be the Medication Adherence Training Interview (i.e., MATI), a structured interview for medication education and adherence training. The MATI has been empirically tested in behavioral medical research in an HIV population and now has been modified for use in patients with NASH. The MATI will be reviewed by study coordinators and used as guidelines for on-going education and addressing medication adherence.

Baseline (day 0)

During this visit the study coordinator will also engage the participant in goal setting. Participants will be asked by the coordinator to set short-term goals (e.g., achieving a near-perfect adherence rate for the next 30 days) that may be important to the efficacy and safety of silymarin therapy. Participants will also be asked to create self-incentives for attaining the stated goals. The rationale is that individuals achieve greater self-directed change if they reward their successful efforts than if they provide no incentives for themselves.

Participants will be given a diary and asked to record the time of day they ingest each dose of study medication. The coordinator will assist the participant with planning a dosing schedule and integrating the medication times into lifestyle patterns. Participants will be encouraged to keep the portable diaries with them at all times or wherever they store their medications in their home or office. Participants will also be asked to track the number of alcoholic beverages consumed on a daily basis. Participants will be encouraged to create a habit of completing the diary on a daily basis, preferably at the time of ingestion.

Study Visits

At subsequent study visits, participants will be asked about tolerance to medications and adherence will be stressed. The participant diary will be reviewed thoroughly and used as a tool to facilitate a discussion about adherence. Coordinators will apply strategies outlined in the original MATI to reinforce adherent behavior, and help participants problem-solve areas that need improvement. The tone of the discussion will be supportive and non-judgmental, while assisting the participant in identifying strategies to improve medication adherence. Coordinators will also review daily alcohol consumption and make recommendations when indicated.

Medication Adherence Outcomes

Primary: Cup counts. Patients will be asked to return all full cups to the coordinators. Missed doses (# of full cups returned) will be counted at each study visit.

Secondary: Patient self-report of missed doses in diaries. Patients will record the time of day for each dose ingestion in diaries. Study coordinators will record the number of self-reported missed doses on a monthly basis. In addition, silymarin levels measured at the specified visits will be used to identify non-adherent patients.

6.0 Statistical methods

Randomization: For the proposed multi-center parallel-arms experimental design with a total of N = 120 patients (30 per Clinical Center), adaptive allocation will be used to minimize the imbalance among treatment arms (overall and within strata). Adaptive allocation will occur by a web-based system, with a manual backup system, devised by the DCC. Participants will be allocated to treatment arm within strata defined by site and diabetes status (yes, no). Adaptive allocation will be performed via a web-based system located in a private and secure area of the server being used for the study. The web-based system will query the user for the information required to identify the stratum and other information to enable checks that the correct patient was assigned (e.g., patient ID, sex) and return the next treatment assignment in that stratum to the user.

Descriptive Analyses

Tabular and graphical descriptive statistical methods will be used to characterize the study sample at baseline in terms of demographic profile severity of disease and clinical measures. These results will be presented for each arm of the study. The numbers of subjects who drop out of the study or have incomplete data will be described. Descriptive statistical methods will also be used to summarize available measures of compliance recorded during the study by dose level.

6.1 Analysis of Safety

All adverse events will be tabulated and summarized by descriptive methods for each arm. Estimates of the toxicity rate for each dose level of silymarin (θ_{420}, θ_{700}) and for placebo (θ_o) will be tabulated along with their 95% confidence intervals.

In safety analyses, the toxicity rates of the three arms of the study will be compared. A test of the null hypothesis H_{o1}: "$\theta_o = \theta_{420} = \theta_{700}$" is not anticipated to be adequately powered due to an anticipated low toxicity rate and findings from the Phase I study, so it is not a primary hypothesis of this phase II study. An idea of the statistical power afforded by the study to the test H_{o1} can be obtained by considering the simplified scenario in which stopping rules for toxicity are not used, and a chi-square test is used to test H_{o1}. In the absence of silymarin, the background rate (θ_o) during the treatment period may be as high as 0.05 (θ_o= 0.05). The maximum tolerated rate is θ_{max} = 0.10. If both of the toxicity rates for silymarin are 0.10, then there is only a 12% chance that data will be collected which yields rejection of H_{o1} at α=0.05.

6.2 Efficacy Analysis

Tabular and graphical descriptive statistical methods will be used to characterize the study group at baseline in terms of demographic and clinical measures. For each arm of the study, the proportion of participants whose NAS is reduced by at least 2 points (π) will be estimated

along with corresponding 95% confidence intervals. Point and interval estimates of the distributions of NAS, ALT, fibrosis score, HOMAr, and at baseline will also be tabulated.

The primary null hypothesis, that the proportion of participants whose NAS is reduced by at least 2 points (H_{o1}: $\pi_o = \pi_1 = \pi_2$) will be tested against the general alternative hypothesis, H_{a1} that response differs in at least one treatment arm via a chi-square test for association with $\alpha = 0.05$. If H_{o1} is rejected, then pairwise comparisons of the arms will be examined using a sequentially rejective test procedure (Holm, 1979). Computation of these results for the ITT analysis will comprise the main results of this study.

A per protocol analysis will also be performed. Participants will be included in this analysis if they took at least 80% of the medication.

The null hypotheses of no difference among treatment arms will be tested for the secondary outcomes. For dichotomous outcomes (secondary outcomes 2 and 3 above), the methods described for testing the primary hypothesis will be used. Change in HOMAr will be compared among the groups using either parametric or non-parametric analysis of variance, as appropriate. The null hypothesis of no treatment differences among the three groups for continuous outcomes will be tested using analysis of variance. Post-hoc tests to identify pairwise differences should the null hypothesis be rejected will control for multiple comparisons.

To maximize statistical power, there will not be an adjustment for the multiple outcomes since the results will be used to guide the design of a proposed phase III study.

6.3 Power considerations

The sample size is determined by the size of the effect (i.e., the percentage of participants whose NAS is reduced by at least 2 points) of silymarin (comparing participants in the placebo arm to those taking silymarin), the type I and II error rates, and the dose response of silymarin.

Assuming that the NAS will be reduced by at least 2 points in 15% of participants in the placebo arm (most likely to occur due to biopsy sampling error or misclassification or because of the lifestyle change) versus 47.5% of participants taking silymarin (40% in the lower dose and 55% in the higher dose group) then with 40 participants in each group will provide 83% power to reject the null hypothesis at α=0.05. For participants who drop out, the last observation carried forward method will be used to impute the final outcome if a follow-up liver biopsy is available. Otherwise the drop out would be considered treatment failures. Thus no over-recruitment to account for loss to follow-up is necessary.

6.4 Pharmacokinetic data analysis

A limited sampling strategy has been devised based on empirical/noncompartmental and model-based approaches. This approach evaluates the family of single point and paired concentrations (observed and interpolated) in order to define the minimal sampling scheme to yield adequate prediction of drug exposure (plasma AUC). A regression-based approach will be utilized using various discrete concentration permutations as predictors and AUC as the response. Based on the availability of a suitable structural model, these results will be validated with a model-based approach based on D-optimal design theory once preliminary data from the Phase I study is available. All modeling and simulations will be performed using the NONMEM software (Version V, Level 1.1, Globomax LLC; Hanover, MD).

Population PK model parameters (fixed and random) will be estimated via nonlinear mixed-effects modeling using NONMEM. An appropriate compartmental model/structure model will be developed for silymarin isomers based on the PK data collected in this study. Data from the Phase I study may be added to improve model stability.The effects of clinical and demographic factors, including but not limited to weight, height, body surface area, body mass index, ALT, age, and gender on PK model parameters will be investigated using a backward stepwise elimination procedure in NONMEM.

Individual predicted PK parameter estimates based on the final model will be used to explore the relationship between various PK metrics and clinical outcomes. Using a logistic regression, the probability of positive (or negative) outcomes will be predicted based on various PK metric expressions (i.e. Cmax, AUC).

6.5 Data Management

Data will be submitted to the DCC via a distributed data entry system. The specifics of the system will be determined by the types and amount of data collected.

DCC personnel will closely monitor clinical center adherence to study protocol and data collection practices to ensure complete and accurate research data. Monitoring will be performed via established data management procedures with on-site monitoring visits conducted at designated intervals or as needed to facilitate the smooth conduct of the study. At the time of the on-site visit, Data Coordinating Center personnel will have access to all study and patient documents as well as availability of clinical center personnel. All patient and study documents will be kept highly confidential.

Data Coordinating Center personnel meet weekly to discuss study status, recruitment, compliance, review data issues or interim analyses, clinical center participation, and other issues that arise during the course of the study.

7.0 Study Organization

7.1 Sites (See Table 1)

This study will be conducted at four clinical centers within the United States. The clinical centers are all tertiary care institutions and academic medical centers with extensive experience in the conduct of clinical trials of antiviral agents for the treatment of chronic hepatitis C. A Data Coordinating Center will coordinate and oversee operations for the study, maintain the database and perform data analyses. This study will use the NIDDK repository. A central virology lab has yet to be selected.

7.2 NIH participants:

NCCAM: Project Scientist – Drs. Qi-Ying Liu and Joshua Berman
NIDDK: Project Scientist- Dr. Edward Doo
NIDDK Consultants: Drs. Jay Hoofnagle and Leonard Seeff

7.3 Committees:

A steering committee and an executive committee were formed to set policy and govern the conduct of the study.

Steering Committee: Serves as the primary governing body of the study; responsible for policy decisions; provides oversight in planning the overall study design, facilitates the conduct and monitoring of the study, and reporting study results; votes on and approves all major decisions, final protocol and subsequent amendments. Members consist of principal investigators of the clinical centers, the coordinating center, and the NCCAM and NIDDK project scientists. A chairperson is appointed by the NIH from among the clinical center PIs.

Executive Committee: Manages day-to-day issues of the study; makes decisions required between the Steering Committee meetings, as needed for efficient progress of the study, and reports its actions to the Steering Committee on a regular basis; organizes and sets agendas for Steering Committee meetings. Members consist of the Steering Committee chair, the Coordinating Center PI, and the NCCAM and NIDDK Project Scientists.

7.4 Subcommittees: Due to the small number of investigators and clinical sites involved in this study, all members will participate on ad hoc committees that will address issues that may arise during the course of the study including measurements, publications & presentations or ancillary studies. In addition, other subcommittees are as follows:

Coordinators Subcommittee: Attend to the day-to-day operations of the study including recruitment, protocol adherence, consistent and complete data collection at each clinical center. Make recommendations to the Steering Committee regarding any study issues that may require modification or resolution.

Exemption Subcommittee:, 2 hepatologists who are not recruiting participants into the study (Drs. Ed Doo and Leonard Seeff, both of the National Institute of Diabetes and Digestive and Kidney Diseases) will review, on a case by case basis, protocol exemption petitions for subjects who do not meet one or more of the eligibility criteria (inclusion and exclusion) but are otherwise considered suitable candidates for enrollment. These exemptions will be considered only when they will not affect primary or secondary endpoints in the study. Examples might include liver biopsies or serological testing outside of predefined windows.

7.5 Data and Safety Monitoring Plan

Introduction

This phase II, randomized controlled trial aims to test the safety and efficacy of silymarin (Legalon®) in participants with NASH. The intervention poses greater than minimal risk to participants. Therefore, the data and safety monitoring plan (DSMP) for this study focuses on close monitoring by the principal investigators (PI) and prompt reporting of excessive adverse events and all serious adverse events to the National Center for Complementary and Alternative Medicine (NCCAM), the National Institute of Diabetes and Digestive and Kidney Diseases (NIDDK), the Data and Safety Monitoring Board (DSMB) and to the participating centers' IRBs.

The Data Coordinating Center (DCC) will monitor clinical center performance (e.g., recruitment, retention, data completeness, timeliness of data collection and submission) and protocol compliance. These reports, with summaries of adverse event data, will be provided to the DSMB for their quarterly reports and biannual calls or meetings, and to the Steering Committee at its annual meeting. DSMB reports will include both open and closed session reports, with only the latter including information by treatment arm.

Safety reports will be sent to the Principal Investigators and the NCCAM and NIDDK Project Officers. The Project Coordinator will be responsible for distributing these reports and assuring that all parties obtain copies of these reports.

The frequency of data review for this study differs according to the type of data and can be summarized in the following table:

Data type	Frequency of review
Recruitment<br>Retention<br>Protocol adherence(e.g., meeting inclusion/exclusion criteria, treatment compliance)	Quarterly reports for DSMB, NCCAM, and NIDDK
Adverse events	Quarterly reports for DSMB, NCCAM and NIDDK
Serious Adverse Events (SAE)	As they occur for NCCAM, NIDDK, and DSMB<br>Quarterly reports for DSMB
Laboratory data	Yearly

Data and Safety Monitoring Board

The DSMB members were appointed by the NCCAM and NIDDK. It consists of 5 members who have no financial, scientific, or other conflict of interest with the study. The DSMB acts as advisors to the NCCAM and NIDDK to monitor participant safety and study progress. The initial responsibility of the DSMB is to review the protocols and approve initiation of the study. Other responsibilities include reviewing informed consent documents; reviewing, commenting and approving the data and safety monitoring plan; assessing data quality, completeness, and timeliness; and evaluating recruitment and retention; monitoring risk versus benefit; consider factors external to the study when relevant information becomes available.

The DSMB will receive all MedWatch forms (FDA Form 3500A) submitted by the clinical centers for serious adverse events.

In the case of a serious adverse event requiring expedited reporting, the Data Coordinating Center will immediately notify Dr. Josh Berman at the NCCAM, holder of the IND, the NCCAM and NIDDK Project Scientists, and the DSMB. For the purposes of this study, reports of all deaths will be expedited, regardless of whether they are considered to be unexpected or related to study drug.

In accordance with IND safety reporting regulations, Dr. Berman, assisted by the Data Coordinating Center, will provide reports of serious adverse events requiring expedited reporting to the appropriate drug review division of the FDA and all participating investigators in writing within 15 calendar days of notification. Reports of serious, unexpected adverse events to the FDA will initially be conducted by telephone or fax within the required time frame (7 calendar days of notification by the clinical centers for telephone and fax safety reports). Following telephone/fax notification, written reports will be sent to the FDA.

The clinical centers are to report serious adverse events to the Data Coordinating Center within 24 hours of knowledge of the event so that notification can be provided as soon as possible following discovery of the event for reasons of patient safety. The clinical center PIs will be responsible for obtaining all information required to complete the adverse event report.

The DSMB will also review appropriate laboratory data annually.

Safety Monitoring

Adverse Events
The clinical centers will be instructed to collect information on all adverse events, defined as:

> "Any unfavorable and unintended sign (including an abnormal laboratory finding, for example), symptom, or disease temporally associated with the use of a medicinal product, whether or not considered related to the medicinal product." (International Conference on Harmonisation of Technical Requirements for Registration of Pharmaceuticals for Human Use, *Guideline for Industry: Clinical SafetyData Management: Definitions and Standards for Expedited Reporting*, March 1995)

Adverse event information will be based on signs or symptoms obtained from the patient or family, during a physical examination, from results of laboratory testing, and clinical evaluation of the patient by the clinical center Principal Investigator (PI) and staff, and will include: the date and time of adverse event occurrence, severity, relation to medication administration, action taken, and outcome.

All serious adverse events, i.e., adverse events that result in, or are, any of the following:

- Death
- Life-threatening
- Inpatient hospitalization or prolongation of existing hospitalization
- Significant or permanent disability
- Congenital anomaly/birth defect
- Medical intervention to prevent permanent damage not requiring inpatient hospitalization
- Development of drug dependency or drug abuse

will be reported to the FDA and other entities (NCCAM, NIDDK, DSMB, IRBs) in cumulative fashion at designated time points (i.e., annual reports will be produced for the FDA and IRB; quarterly reports will be produced for the DSMB, NCCAM and NIDDK).

If the patient experiences a Serious Adverse Event:

1. Clinical center personnel will complete the FDA MedWatch form.
2. The completed MedWatch form must be faxed to the Coordinating Center at the University of Pittsburgh within 24 hours of knowledge of the event.
3. The Coordinating Center will submit the MedWatch form to the chair of the DSMB.
4. Dr. Berman holder of the IND, will report SAEs that meet criteria for expedited reporting to the FDA.

The Clinical Center Principal Investigators will be responsible for notifying their local Institutional Review Board.

Adverse Events Requiring Expedited Reporting

An adverse event will be subject to expedited reporting if it meets all of the following criteria, which are defined and discussed in detail below:

1) Serious in nature
2) Related to medicinal product
3) Unexpected

All deaths will be subject to expedited reporting, regardless of whether they are considered unexpected or related to medicinal product.

Related to Medicinal Product

The phrase "**related to the medicinal product**" implies causality or attributability to Legalon®. An adverse event should be considered to be related to Legalon® in situations in which a causal relationship cannot be ruled out. As noted above, the clinical center PIs will assess the degree of relatedness of the study medications and adverse events (i.e., unrelated, possibly related, probably related, definitely related).

Unexpected

The term **unexpected** refers to an adverse event that has not been previously observed or documented. A guideline is needed to define an adverse event as either expected or unexpected based on previous observation. The following documents or circumstances will be used to determine the expectedness of an adverse event:

- The Drug Label which contains the clinical and non-clinical data on therapy with Legalon®.
- The natural history of NASH.
- Reports, which add significant information on specificity or severity of an otherwise known and documented adverse event, associated with the use of Legalon®. Thus more severe or more specific adverse events than had previously been observed are considered unexpected.

Expedited reporting is also required when there is an increased rate of occurrence of expected, serious adverse events related to the medicinal product. Hence, the DCC will prepare reports of rates of serious adverse events for review at DSMB calls and meetings.

Sufficient data on adverse events requiring expedited reporting must be obtained by clinical center personnel to enable clinical center personnel to complete the MedWatch form (FDA Form 3500A).

The DCC will send copies of the initial notification and written reports on adverse events to the NCCAM and NIDDK project scientists, Dr. Berman, the DSMB, the other clinical centers, and Rottapharm/Madaus. The Data Coordinating Center Principal Investigator, project coordinator, and data managers will be notified of serious adverse events.

All adverse event information will be included in the annual IND report to the FDA. The initial annual report will be within 60 days of the anniversary date that the IND went into effect. In addition, the Data Coordinating Center will provide the clinical centers with updated information on adverse events that occur throughout the study. This information may be used to change the protocol or consent form if necessary. The clinical centers will also need to provide this information to their respective IRB offices annually with their renewals, or in the timeframes required by their IRBs.

The FDA annual report will be prepared by the DCC with input from the clinical investigators.

Stopping rules

There will be no planned interim analysis. Safety information will be examined by the DSMB (frequency TBD by DSMB).

Participant Confidentiality

The central database of the study is on a server at the Epidemiology Data Center (EDC) in the Graduate School of Public Health at the University of Pittsburgh secured behind locked doors and an alarm with password access provided only to authorized personnel. Backups are performed daily to guard against data loss due to an equipment or power failure. Scheduled backups and archives at the EDC protect central and local information from hard disk failures. Tape backup volumes and CD-ROM copies of critical project files are located in a secured off-site storage area to prevent data loss due to catastrophic events. Routine virus detection is also enforced for all EDC computers involved in the study. All critical information regarding database transactions is logged and stored in journal files. In the event of accidental corruption of the project database, a previous database state may be restored from backup volumes or journal files. All servers used for this project are connected to uninterrupted power supplies to protect equipment against electrical surges and outages. A secured, raised-floor computer room in an area with a burglar alarm houses all project server equipment.

Subject confidentiality is preserved by assigning alphanumeric subject IDs at the clinical centers. Data sent to the DCC are identified by alphanumeric ID only. No reports of this study will use names or other identifying information such as social security numbers or addresses. Data, with alphanumeric ID only, will be stored at the DCC indefinitely. In addition, data and biospecimens, with alphanumeric ID only, will be stored indefinitely in the NIDDK data archives and biospecimen repository and may be used for future research.

LITERATURE CITED

[1] Ludwig J, Viggiano RT, McGill DB, Oh BJ. Nonalcoholic steatohepatitis: Mayo Clinic experiences with a hitherto unnamed disease. Mayo Clini Proc 1980;55:434-438.

[2] Neuschwander-Tetri B, Caldwell S. Nonalcoholic steatohepatitis: summary of an AASLD single topic conference. Hepatology 2003;37:1202-1219

[3] Angulo P, Keach JC, Batts KP, Lindor KD. Independent predictors of liver fibrosis in patients with nonalcoholic steatohepatitis. Hepatology 1999;30:1356-1362

[4] Matteoni CA, Younossi ZM, Gramlich T, Boparai N, Liu YC, McCullough AJ. Nonalcoholic fatty liver disease: a spectrum of clinical and pathological severity. Gastroenterology 1999;116:1413-1419.

[5] Bacon BR, Farahvash MJ, Janney CG, Neuschwander-Tetri BA. Nonalcoholic steatohepatitis: an expanded clinical entity. Gastroenterology 1994;107:1103-1109.

[6] Santos L, Molina EG, Jeffers LJ, Reddy KR, Schiff ER. Prevalence of nonalcoholic steatohepatitis among ethnic groups (Abstract). Gastroenterology 2001;120:A117.

[7] Weston SR. Leyden W. Murphy R. Bass NM. Bell BP. Manos MM. Terrault NA. Racial and ethnic distribution of nonalcoholic fatty liver in persons with newly diagnosed chronic liver disease. Hepatology 2005; 41:372-379.

[8] Portincasa P, Grattagliano I, Palmieri V, Palasciano G. Nonalcoholic steatohepatitis: recent advances from experimental models to clinical management. Clinical Biochemistry 2005;38:203-217.

[9] Havel P. Section IV: Lipid Modulators of Islet Function Update on Adipocyte hormones regulation of energy balance and carbohydrate/lipid metabolism. Diabetes 2004;53:S143-S151.

[10] Hui J, Hodge A, Farrell G, Kench J, Kriketoa A, George J. Beyond insulin resistance in NASH: TNF-α or adiponectin? Hepatology 2004;40:46-54.

[11] Brunt EM. Nonalcoholic steatohepatitis. Semin Liver Dis 2004;24:3-20.

[12] Kleiner DE. Brunt EM. Van Natta M. Behling C. Contos MJ. Cummings OW. Ferrell LD. Liu YC. Torbenson MS. Unalp-Arida A. Yeh M. McCullough AJ. Sanyal AJ. Nonalcoholic Steatohepatitis Clinical Research Network. Design and validation of a histological scoring system for nonalcoholic fatty liver disease. Hepatology. 2005;41:1313-21.

[13] Brunt E, Janney C, Di Bisceglie A, Neuschwander-Tetri B, Bacon B. Nonalcoholic steatohepatitis: a proposal for grading and staging the histological lesions. The American Journal of Gastroenterology 1999;94:2467-2474.

[14] Ratziu V, Charlotte, F, heartier S, Gombert S, Giral P, Bruckert E, Grimaldi A, Capron F, Poynard T. Sampling variability of liver biopsy in nonalcoholic fatty liver disease. Hepatology 2005;128:1898-1906.

[15] Neuschwander-Tetri B, Brunt E, Wehmeier K, Oliver D, Bacon B. Improved nonalcoholic steatohepatitis after 48 weeks of treatment with PPAR-γ ligand rosiglitazone. Hepatology 2003;38:1008-1017.

[16] Promrat K. Lutchman G. Uwaifo GI. Freedman RJ. Soza A. Heller T. Doo E. Ghany M. Premkumar A. Park Y. Liang TJ. Yanovski JA. Kleiner DE. Hoofnagle JH. A pilot study of pioglitazone treatment for nonalcoholic steatohepatitis. Hepatology. 2004;39:188-96.

[17] Kvasnicka F, Biba B, Sevcik R, Voldrich M, Kratka J. Analysis of the active components of silymarin. Journal of Chromatography A 2003;990:239-245.

[18] Jacobs B, Dennehy C, Ramirez G, Snapp J, Lawrence V. Milk thistle for the treatment of liver disease: a systematic review and meta-analysis. The American Journal of Medicine 2002;113:506-515.

[19] Pepping J. Milk thistle: silybum marianum: alternative therapies. American Journal of Health-System Pharmacy 1999;56:1195-1197.

[20] Lorenz D, Lucker PW, Mennicke WH, Wetzelberger N. Pharmacokinetic studies with silymarin in human serum and bile. Meth and Find Exptl Clin Pharmacol 1984;6:655-661.

[21] Orlando R, Fragasso A, Lampertico M, Marena C. Silybin kinetics in patients with liver cirrhosis: a comparitive study of a silybin-phospatidylcholine complex (Siliphos®) and silymarin. Med. Sci Res. 1990;18:861-863.

[22] Sridar C, Goosen TC, Kent UM, Williams JA, Hollenberg PF Silybin inactivates cytochromes P450 3A4 and 2C9 and inhibits major hepatic glucuronosyltransferases Drug Metab Dispos. 2004;32:587-94.

[23] D'Andrea V, Perez LM, Sanchez Pozzi EJ. Inhibition of rat liver UDP-glucuronosyltransferase by silymarin and the metabolite silibinin-glucuronide. Life Sci. 2005;77:683-92.

[24] Zhang S, Morris ME. Effects of the flavonoids biochanin A, morin, phloretin, and silymarin on P-glycoprotein-mediated transport. J Pharmacol Exp Ther. 2003;4:1258-67.

[25] Chung SY, Sung MK, Kim NH, Jang JO, Go EJ, Lee HJ. Inhibition of P-glycoprotein by natural products in human breast cancer cells. Arch Pharm Res 2005;28:823-8.

[26] Patel J, Buddha B, Dey S, Pal D, Mitra AK. In vitro interaction of the HIV protease inhibitor Ritonavir with herbal constituents: changes in P-gp and CYP3A4 activity Am J Ther 2004;11:262-77.

[27] Mulrow C, Lawrence V, Jacobs B, Dennehy C, Sapp J, Ramirez G, Aguilar C, Montgomery K, Mobidoni L, Arterburn JM, Chiquette E, Harris M, Mullins D, Vickers A, Flora K. Milk thistle: effects on liver disease and cirrhosis and clinical adverse effects. Evidence Report/Technology Assessment No. 21. AHRQ Publication No. 01-E025. Rockville, MD: Agency for Healthcare Research and Quality. October 2000.

[28] Adverse Drug Reactions Advisory Committee. An adverse reaction to the herbal mediciation milk thistle (silybum marianum). MJA 1999;170:218-219.

[29] Saller R, Meier R, Brignoli R. The use of silymarin in the treatment of liver diseases. Drugs 2001;61:2035-2061.

[30] Bosisio E, Benelli C, Pirola O. Effect of flavanolignans of silybum marianum L. on lipid peroxidation in rat liver microsomes and freshly isolated hepatocytes. Pharmacol Res 1992;25:147-54.

[31] Carini R, Comoglio A, Albano E, Poli G. Lipid peroxidation and irreversible damage in the rat hepatocyte model: protection by the silybin-phospholipid complex IdB 1016. Biochemical Pharmacology 1992;43:2111-2115.

[32] Mira L, Silva M, Manso CF. Scavenging of reactive oxygen species by silibinin dihemisuccinate. Biochem Pharamacol 1994;42:964-8.

[33] Flora K, Hahn M, Rosen H, Benner K. Milk thistle (s*ilybum marianum*) for the therapy of liver disease. The American Journal of Gastroenterology 1998;93:139-143.

[34] Lirussi F. Okolicsanyi L. Cytoprotection in the nineties: experience with ursodeoxycholic acid and silymarin in chronic liver disease. Acta Physiologica Hungarica 1992;80:363-7.

[35] Buzzelli G. Moscarella S. Giusti A. Duchini A. Marena C. Lampertico M. A pilot study on the liver protective effect of silybin-phosphatidylcholine complex (IdB1016) in chronic active hepatitis. International Journal of Clinical Pharmacology, Therapy, & Toxicology 1993;31:456-60.

[36] Ferenci P. Dragosics B. Dittrich H. Frank H. Benda L. Lochs H. Meryn S. Base W. Schneider B. Randomized controlled trial of silymarin treatment in patients with cirrhosis of the liver. Journal of Hepatology 1989;9:105-13.

[37] Pares A. Planas R. Torres M. Caballeria J. Viver JM. Acero D. Panes J. Rigau J. Santos J. Rodes J. Effects of silymarin in alcoholic patients with cirrhosis of the liver: results of a controlled, double-blind, randomized and multicenter trial. Journal of Hepatology. 1998;28:615-21.

[38] Velussi M. Cernigoi AM. De Monte A. Dapas F. Caffau C. Zilli M. Long-term (12 months) treatment with an anti-oxidant drug (silymarin) is effective on hyperinsulinemia, exogenous insulin need and malondialdehyde levels in cirrhotic diabetic patients. Journal of Hepatology. 1997;26:871-9.

[39] Huber R, Futter I, Ludtke R. Oral Silymarin for chronic hepatitis C - a retrospective analysis comparing three dose regimens. Eur J Med Res 2005;10:68-70.

[40] Venkataramanan R, Ramachandran V, Komoroski BJ, Zhang S, Schiff PL, Strom SC. Milk thistle, a herbal supplement, decreases the activity of CYP3A4 and uridine diphosphoglucuronosyl transferase in human hepatocyte cultures. Drug Metab Dispos 2000;28:1270-3.

[41] Beckmann-Knopp S, Rietbrock S, Weyhenmeyer R, Bocker RH, Beckurts KT, Lang W, Hunz M, Fuhr U. Inhibitory effects of silibinin on cytochrome P-450 enzymes in human liver microsomes. Pharmacol Toxicol 2000;86:250-6.

[42] Zuber R, Modriansky M, Dvorak Z, Rohovsky P, Ulrichova J, Simanek V, Anzenbacher P. Effect of silybin and its congeners on human liver microsomal cytochrome P450 activities. Phytother Res 2002;16:632-8.

[43] Gurley BJ, Gardner SF, Hubbard MA, Williams DK, Gentry WB, Carrier J, Khan IA, Edwards DJ, Shah A. In vivo assessment of botanical supplementation on human cytochrome P450 phenotypes: Citrus aurantium, Echinacea purpurea, milk thistle, and saw palmetto. Clin Pharmacol Ther 2004;76:428-40.

[44] DiCenzo R, Shelton M, Jordan K, Koval C, Forrest A, Reichman R, Morse G. Coadministration of milk thistle and indinavir in healthy subjects. Pharmacotherapy 2003;23:866-70.

[45] Piscitelli SC, Formentini E, Burstein AH, Alfaro R, Jagannatha S, Falloon J. Effect of milk thistle on the pharmacokinetics of indinavir in healthy volunteers. Pharmacotherapy 2002;22:551-6.

[46] Leber HW, Knauff S. Influence of Silymarin on drug metabolizing enzymes in rat and man. Arzneimittelforschung 1976;26:1603-5.

[47] Raucy JL. Regulation of CYP3A4 expression in human hepatocytes by pharmaceuticals and natural products. Drug Metab Dispos 2003;31:533-9.

[48] Flaig et al. A Phase I and pharmacokinetic study of silybin-phytosome in prostate cancer patients. Invest New Drugs 2007;25:139-46.

[49] Schoeller,DA But how much physical activity? American Journal of Clinical Nutrition 2003;78:669-70.

Appendix 1 = SyNCH Phase I- Pharmacokinetic Summary & Tables
Appendix 2 = Phase I Safety Summary Report
Appendix 3 = IND
Appendix 4 = Commom Toxicity Criteria for Adverse Events (CTCAE) v3.0
Appendix 5 = Time Event Table
Appendix 6 = Guidelines for diet and exercise
Appendix 7 = Pathology Scoring System
